# Facet- and Gas-Dependent Reshaping of Au Nanoplates by Plasma Treatment

**DOI:** 10.1021/acsnano.1c00861

**Published:** 2021-06-11

**Authors:** Ruoqi Ai, Christina Boukouvala, George Lewis, Hao Wang, Han Zhang, Yunhe Lai, He Huang, Emilie Ringe, Lei Shao, Jianfang Wang

**Affiliations:** † Department of Physics, The Chinese University of Hong Kong, Shatin, Hong Kong SAR China; ‡ Department of Materials Science and Metallurgy, University of Cambridge, Cambridge CB3 0FS, United Kingdom; § Department of Earth Sciences, University of Cambridge, Cambridge CB2 3EQ, United Kingdom; # Shenzhen JL Computational Science and Applied Research Institute, Shenzhen 518109, China

**Keywords:** gold nanocrystals, gold nanoplates, plasma treatment, plasmon resonance, reshaping, surface-enhanced Raman scattering

## Abstract

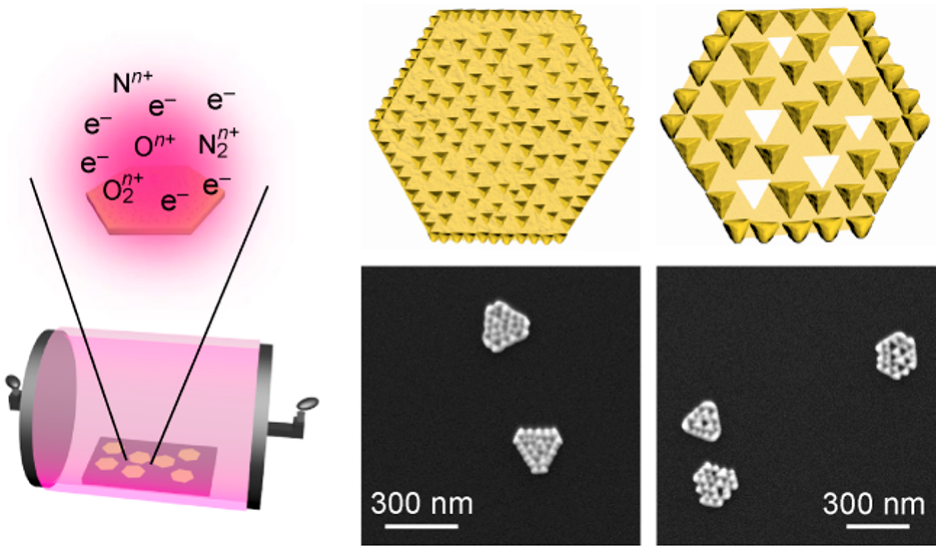

The reshaping of metal nanocrystals on substrates is usually realized by pulsed laser irradiation or ion-beam milling with complex procedures. In this work, we demonstrate a simple method for reshaping immobilized Au nanoplates through plasma treatment. Au nanoplates can be reshaped gradually with nearly periodic right pyramid arrays formed on the surface of the nanoplates. The gaseous environment in the plasma-treatment system plays a significant role in the reshaping process with only nitrogen-containing environments leading to reshaping. The reshaping phenomenon is facet-dependent, with right pyramids formed only on the exposed {111} facets of the Au nanoplates. The morphological change of the Au nanoplates induced by the plasma treatment leads to large plasmon peak redshifts. The reshaped Au nanoplates possess slightly higher refractive index sensitivities and largely increased surface-enhanced Raman scattering intensities compared to the flat, untreated nanoplates. Our results offer insights for studying the interaction mechanism between plasma and the different facets of noble metal nanocrystals and an approach for reshaping light-interacting noble metal nanocrystals.

Noble metal nanocrystals confine light into the subwavelength scale and enhance light–matter interactions because of their localized surface plasmon resonances (LSPRs). The LSPR energy is highly tailorable by controlling the nanocrystal material, size, shape, the surrounding environment, the spacing, and geometry of assembled metal nanocrystals.^1−4^ Because of their highly adjustable LSPRs and attractive LSPR-induced optical properties, a wide range of applications has been demonstrated for noble metal nanocrystals, such as photocatalysis,^5,6^ sensing,^7,8^ imaging,^9^ surface-enhanced spectroscopy,^10^ photothermal therapy,^11^ and color display.^12−14^

Many of these applications involve the deposition of metal nanocrystals on various substrates for simple support or for the design of functional plasmonic nanostructures and devices.^15−18^ In most cases, the shape and size of metal nanocrystals remain the same as from preparation. There are also situations where metal nanocrystals are further reshaped after they are deposited on substrates. For example, thermal treatment can transform Au nanorods into nanospheres, leading to tailorable longitudinal plasmon resonance properties.^19,20^ Focused-ion beam (FIB) has been used to cut metallic nanostructures into different shapes. In one example, helium-ion beam milling can cut near-perfect metallic spheres produced by a laser-induced transfer method and make split-ball nanoresonators.^21^ In another example, FIB can induce the folding of three-dimensional (3D) nanostructures to give plasmonic “nanograters”, which exhibit interesting 3D hybridization in current flow and Fano resonance.^22^ In addition, pulsed laser light has been used to shape the continuous metal surface into a variety of nanostructures, such as nanojets,^23^ nanobumps,^24^ ripples,^25^ nanodroplets^26^ and cavities.^27,28^ Single laser pulses can also transform individual anisotropic nanocrystals into nanospheres due to the high instant energy.^12−14,29^ The change of the shape shifts the plasmon resonance wavelength, leading to the variation in the reflected color.^12−14^ The above fabrication methods rely on high-energy ions and photons to process the surface of the material, either through direct milling or indirect heating upon photothermal conversion.

In addition to energetic ions and photons, plasma, which is composed of ions and free electrons, can be produced artificially by heating a neutral gas or by subjecting a gas to a strong electromagnetic field. Plasma treatment has been extensively used for removing contaminants from^30^ and modifying the surfaces^31^ of various substrates. O_2_ or Ar plasma can be used to modify a surface to increase its roughness and render it hydrophilic or hydrophobic.^32,33^ Moreover, O_2_ plasma with high energy can break most bonds of surface organic contaminants, such as C–H and C–C, and provide different active species reacting with organic contaminants to clean the surfaces of inorganic nanocrystals. For instance, plasma treatment has been employed to remove the stabilizing organic molecules from noble metal nanocrystals.^34,35^ However, to date, there have been no reports on the use of plasma treatment to reshape noble metal nanocrystals.

Herein we report on the use of plasma treatment to reshape colloidal Au nanoplates (NPLs) that are presynthesized through seed-mediated growth and deposited on substrates. Plasma treatment causes the top surface of the Au NPLs to become rough and patterned with right pyramids. With the increase in the duration of plasma treatment, each Au NPL becomes thinner, the right pyramids on its top surface grow larger, and the number of the pyramids is gradually reduced. The reshaping phenomenon has been found to occur only in the presence of nitrogen. Moreover, the reshaping process is facet-dependent. It can only occur on the {111} facets of the Au NPLs. During the reshaping process, the plasmon peak of the Au NPLs gradually redshifts. Furthermore, the reshaped Au NPLs can induce stronger surface-enhanced Raman scattering (SERS) signals than the original, flat Au NPLs. Our results offer an approach for the reshaping of pregrown plasmonic Au nanocrystals deposited on substrates. They will also stimulate further studies on the fundamental interaction of atomic and molecular species with the different crystalline facets of noble metal nanocrystals.

## Results and Discussion

The Au NPL samples with different thicknesses were grown by a seed-mediated method using cetyltrimethylammonium bromide (CTAB) as the stabilizing agent, as reported previously.^36^ By adding different amounts of the Au precursor, five Au NPL samples with different thicknesses ranging from ∼19 to ∼67 nm were prepared. All of the Au NPLs show an approximately hexagonal shape. Their lateral sizes were measured to be ∼170 nm from their scanning electron microscopy (SEM) images (Figure S1a). The extinction spectra of the Au NPL samples display both the in-plane dipole plasmon peak and the in-plane quadrupole plasmon peak (Figure S1b). The dipole and quadrupole plasmon peaks blueshift from 1056 to 752 nm and from 720 to 590 nm, respectively, as the NPL thickness is increased. The surfaces of the NPLs are atomically flat. The average thicknesses of the five samples are 19 ± 2, 31 ± 1, 41 ± 2, 53 ± 4, and 67 ± 5 nm, which were measured by atomic force microscopy (AFM) (Figure S2).

**Figure 1:**
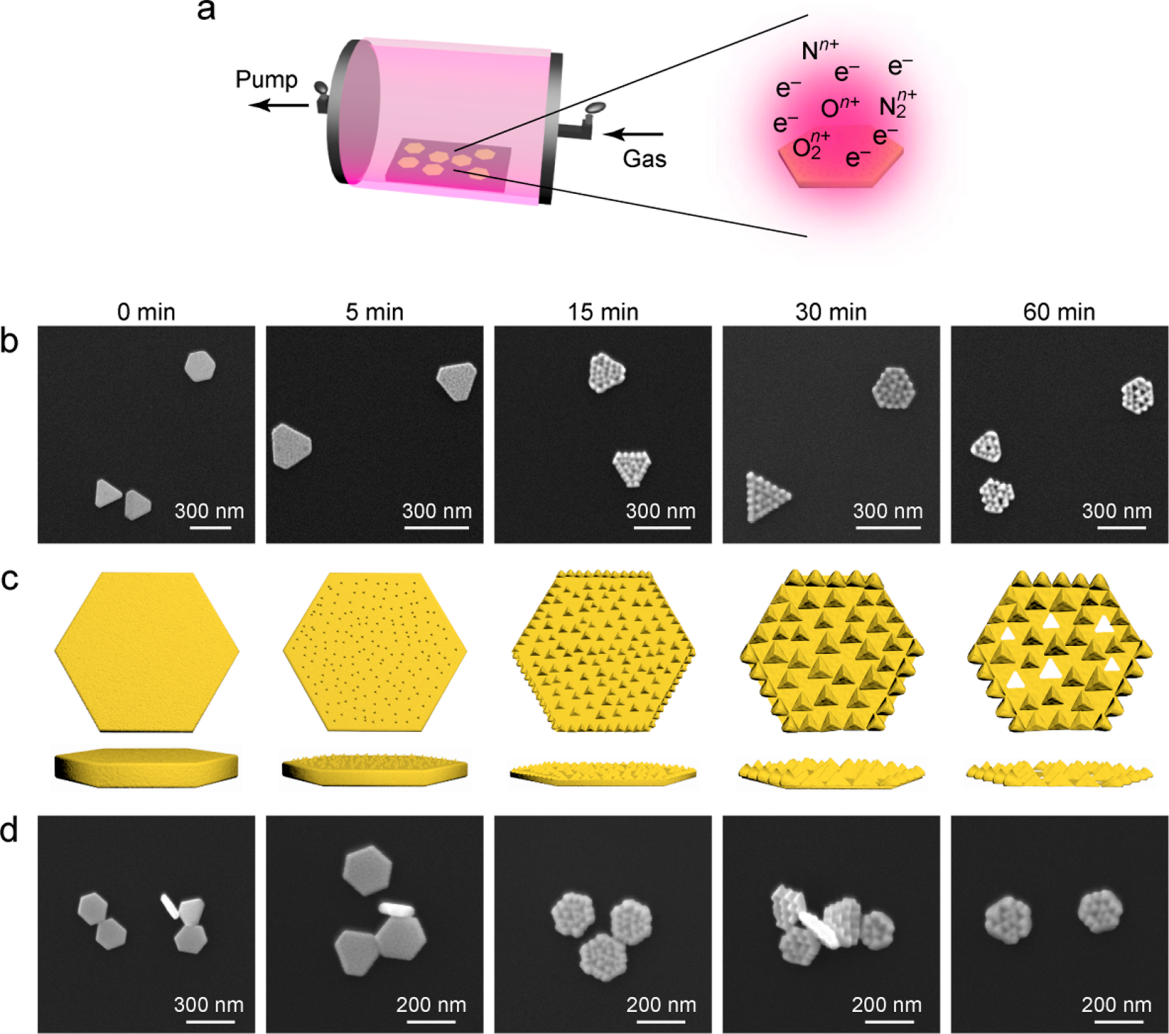
Au NPL surface reshaping. (a) Schematic showing the plasma treatment of Au NPLs. (b) SEM images of the 19 nm thick Au NPL sample after the plasma treatment in air for 0, 5, 15, 30, and 60 min. (c) Schematics illustrating the Au NPLs plasma-treated for increasing periods of time. (d) SEM images of the 41 nm thick Au NPL sample after the plasma treatment in air for 0, 5, 15, 30, and 60 min. The medium plasma power (10.5 W) was used.

The as-prepared Au NPL samples were deposited on Si substrates, followed by the treatment in a plasma cleaner at a set power. The chamber was prefilled with air and pumped down to ∼0.3 mbar. A glow composed of a mixture of ionized gaseous atomic and molecular species and electrons was generated in the chamber (Figure 1a). The substrate was treated for different periods of time in air plasma. The surfaces of the Au NPLs were dramatically reconstructed. The top surface of the Au NPLs was covered with a nearly periodic array of right pyramids after the air plasma treatment (Figure 1b). The three exposed surfaces of each right pyramid are the {100} facets (see the discussion below). With increasing treatment time, the thickness of each Au NPL decreases, the number of the right pyramids on the top surface of the NPL decreases, and the size of each right pyramid increases, as schematically illustrated in Figure 1c. The morphological evolution was also examined by AFM (Figure S3). As the treatment time is increased, the top of the pyramids becomes higher in comparison with the thickness of the untreated Au NPLs. Moreover, the Au NPLs can become perforated if the NPLs are sufficiently thin, as shown in Figure 1b for the NPLs with an average thickness of 19 nm. The air plasma treatment was also performed on other Au NPL samples with the thicknesses of 31, 41, 53, and 67 nm (Figure 1d and Figure S4). All Au NPLs exhibit similar time-dependent morphological evolution upon the air plasma treatment except that the thicker ones are difficult to be perforated. Since both the top and bottom flat surfaces of the Au NPLs are {111} facets,^36,37^ it seems that air plasma can cause the reconstruction of the {111} facets and the production of right pyramids on the top surface of the Au NPLs. The effect of the power on the plasma treatment was also investigated. The 19 nm thick Au NPL sample was treated in air plasma for 30 min at low (6.8 W), medium (10.5 W), and high (18 W) power levels, respectively (Figure S5). The extent of the surface reshaping increases with the plasma power. When the plasma power is low, the top surface of the NPLs is only slightly modified. The change of the surface morphology becomes more significant at the medium power, as indicated by the enlarged right pyramids. After the high-power plasma treatment for 30 min, the Au NPLs are perforated, and the right pyramids become larger and well-developed. In the following experiments, the plasma power was fixed at the medium level.

**Figure 2:**
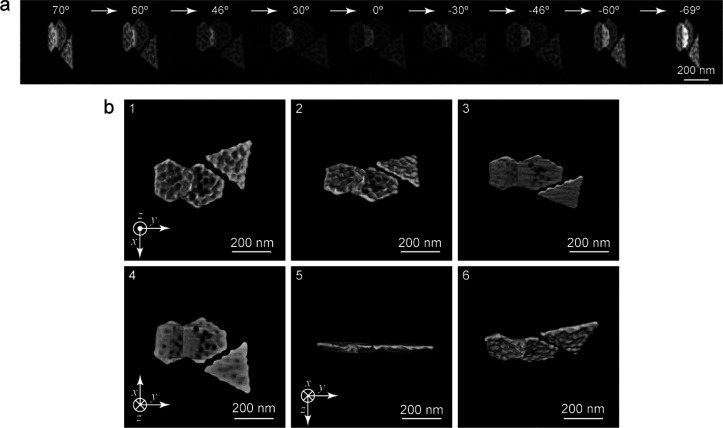
Electron tomography of three reshaped Au NPLs. (a) HAADF STEM images across the tilt series. The tilt axis is vertical in the page. (b) Reconstructed images of the three Au NPLs. The NPLs are rotated around the *y*-axis in the images from 1 to 6. The Au NPLs were treated in air plasma for 30 min.

To better observe the morphologies of the plasma-treated Au NPLs, 3D electron tomography based on high-angle annular dark-field scanning transmission electron microscopy (HAADF STEM) imaging was performed. The Au NPLs were deposited on TEM grids made of Si and with windows covered with an electron beam-transparent thin Si_3_N_4_ film and treated in air plasma. Figure 2a shows the raw HAADF STEM data of three 19 nm thick Au NPLs treated in air plasma for 30 min. Two Au NPLs are slightly overlapped. The particles are tilted around an axis vertical to the page during the tomography acquisition. Reconstructed volumes, shown in Figure 2b, reveal that the top surfaces (exposed to plasma) are reshaped dramatically to give right pyramids, while the bottom surfaces (in contact with the Si_3_N_4_ surface) are still flat and remain nearly intact. The latter can be ascribed to the intimate contact between the Au NPLs and the Si_3_N_4_ film, both flat, preventing penetration of the plasma. In addition, some holes are seen reaching the bottom side. They confirm the perforations caused by the plasma treatment. Similar 3D tomography visualization results, obtained on a NPL treated in air plasma for 30 min, are reported in Figure S6. In addition, tomography was performed on the Au NPLs treated in air plasma for 5 min (Figure S7), showing only slight modification of the top surfaces and the absence of well-defined pyramids. Their bottom surfaces also appear flat. Taken together, the electron tomography results verify the reshaping induced by the plasma treatment in air and its dependence on the plasma treatment time. The reshaping occurs mainly on the top surfaces of the Au NPLs that are supported on flat substrates.

**Figure 3:**
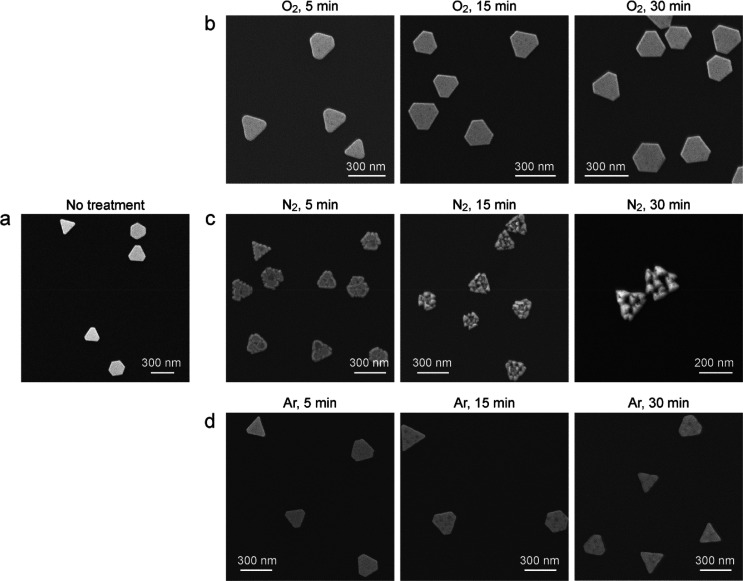
Au NPLs subjected to the plasma treatments in different gaseous environments. (a) SEM image of the 19 nm thick Au NPLs without plasma treatment. (b–d) SEM images of the Au NPLs plasma-treated in O_2_, N_2_, and Ar, respectively, for different periods of time.

The plasma treatment was next carried out on the Au NPLs in different gaseous environments. The Au NPL sample with an average thickness of 19 nm was employed (Figure 3a). The morphological changes of the Au NPLs were examined by SEM imaging after the plasma treatments in different gaseous environments. The surfaces of the Au NPLs are barely changed in the pure O_2_ environment (Figure 3b). In contrast, the top surfaces are clearly modified in the pure N_2_ environment and are also covered with right pyramids (Figure 3c). With the increase of the plasma-treatment time, the Au NPLs become thinner and the right pyramids on the top surfaces grow larger. Compared with the air plasma treatment (Figure 1b), the pure N_2_ plasma treatment produces larger right pyramids and greater morphological changes on the Au NPLs with the same treatment time. As a result, pure N_2_ plasma exhibits a stronger reshaping capability toward the Au NPLs than air plasma. In comparison, after being treated in Ar plasma for 1 h, the Au NPLs show slightly rounded corners. The shape of the Au NPLs is maintained without the generation of right pyramids (Figure 3d). The rounding of the Au NPLs can be attributed to atom migration caused by the heat from the plasma treatment. Figure S8 shows the similar results for the 41 nm thick Au NPLs plasma-treated in the different gaseous environments. The Au NPLs can be reshaped in both air and pure N_2_ environments, while O_2_ or Ar plasma has no reshaping effect on the Au NPLs. Taken together, the results from the plasma treatments in the different gaseous environments indicate that N_2_ plasma plays a critical role in the reshaping of the Au NPLs. It is N_2_ molecules contained in air that take effect in the air plasma treatment.

**Figure 4:**
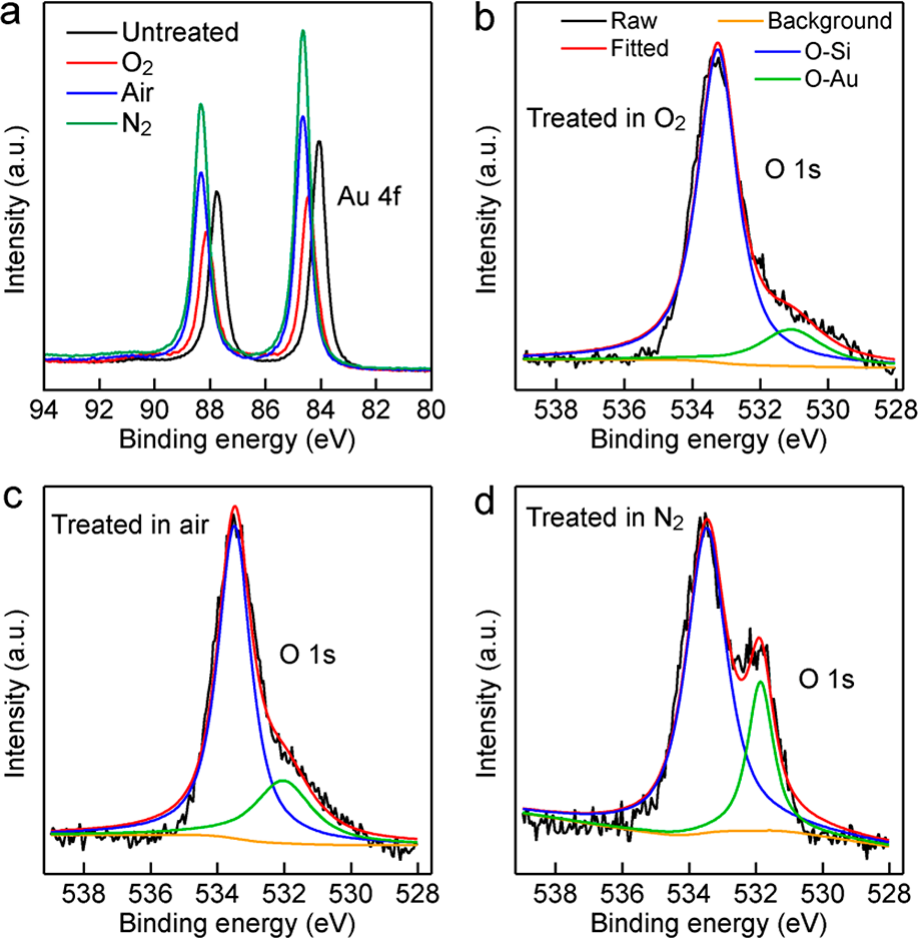
XPS spectra of the 53 nm thick Au NPLs treated in the different gaseous environments. (a) Au 4f peak. (b) O 1s peak after the O_2_ plasma treatment. (c) O 1s peak after the air plasma treatment. (d) O 1s peak after the N_2_ plasma treatment.

The top surfaces of the Au NPLs belong to the {111} facets. They can be reshaped in air and pure N_2_ environments. The chemical state of the surface Au atoms might be changed after the reshaping process. In addition, there might be atomic and molecular species adsorbed on the Au surface. To examine the chemical states on the surfaces of the Au NPLs plasma-treated in the different gaseous environments, X-ray photoelectron spectroscopy (XPS) measurements were performed on four 53 nm thick Au NPL samples, which are the untreated, pure O_2_ plasma-treated, air plasma-treated, and pure N_2_ plasma-treated. The Au 4f spectrum of the untreated sample is composed of the Au 4f_7/2_ at 84.8 eV and 4f_5/2_ at 87.7 eV (Figure 4a). In comparison with the untreated sample, both Au 4f_7/2_ and 4f_5/2_ peaks of the plasma-treated samples are shifted toward the higher binding energies. This is probably caused by the loss of electrons from the surface Au atoms through oxidation during the plasma treatments. The Au 4f binding energies of the N_2_ and air plasma-treated Au NPL samples are nearly identical, and larger than those of the pure O_2_ plasma-treated one. This result suggests that N_2_ plasma can cause electron loss on the surface Au atoms. The O 1s XPS spectrum of the untreated Au NPLs shows only one peak (Figure S9), which can be attributed to the O–Si bond due to the presence of silicon oxide on the Si substrate. The O 1s XPS peaks of the Au NPLs treated in O_2_, air and N_2_ split into two peaks (Figure 4b–d). The higher binding energy peak is originated from O–Si. The lower binding energy peak can be assigned to O–Au. Furthermore, as the gaseous environment contains more N_2_, the integrated peak area of O–Au increases. This increase is probably caused by more surface Au atoms losing electrons and more O–Au bonds being formed during the plasma treatment. However, no N 1s signal can be detected by XPS (Figure S10), implying that no N-containing species are adsorbed on the Au surface after any plasma treatment. More experimental and theoretical studies are required to fully understand how N_2_ plasma causes the adsorption of O-containing species on the Au surface and how the (111) facets are reconstructed upon the N_2_ plasma treatment.

To investigate if the N_2_ plasma-induced reshaping is dependent on the specific facets of Au nanocrystals, three more differently shaped Au nanocrystal samples were synthesized (Figure S11). They include Au nanobipyramids (NBPs), Au nanocubes (NCs), and Au nanorods (NRs). All of them were synthesized by seed-mediated growth methods, using CTAB and cetyltrimethylammonium chloride as the stabilizing and structure-directing agents.^38−40^ The Au NCs are encapsulated with the {100} facets.^41^ The side facets of the Au NBPs are {11*n*} with *n* being an integer and dependent on the tip angle.^42^ The side surfaces of the Au NRs are composed of the {100} and {110} facets, and the ends are encapsulated with high-index facets, which are dependent on the specific shape at the ends.^43^ In comparison, the flat top and bottom surfaces of the hexagonal Au NPLs are the {111} facets, and the side surfaces of the hexagonal NPLs are composed of the alternating {111} and {100} facet pairs.^36^ The three Au nanocrystal samples were deliberately mixed together with the Au NPLs and treated with air plasma. After the air plasma treatment, only the {111} facets of the Au NPLs are reconstructed with the appearance of right pyramids on the top surfaces (Figure S12). There are nearly no changes on the other facets that are associated with the Au NCs, NRs, and NBPs. With the increase in the plasma-treatment duration, the ends of the Au NRs and NBPs and the corners of the Au NCs become slightly rounded. The slight rounding can be attributed to the temperature rise caused by the plasma treatment. These results show that the air plasma treatment can only cause the reconstruction of the {111} facets to generate right pyramids. We further synthesized three Au NR samples with different aspect ratios and treated them in air and N_2_ plasma. The average lengths and diameters of the NR samples are 89 ± 6 nm/22 ± 2 nm, 102 ± 7 nm/30 ± 3 nm, and 139 ± 11 nm/56 ± 6 nm, respectively (Figure S13). All of the Au NRs remain unchanged in shape after the air plasma treatment for 30 min (Figure S14). However, the ends and edges of the Au NRs become rounded and the Au NRs become slightly shorter, especially for the smallest NR sample, after the N_2_ plasma treatment for 30 min. The difference in the reshaping behavior between the air and N_2_ plasma treatments can be ascribed to the fact that the chemically grown Au NRs have slightly truncated edges and corners. There exist tiny exposed {111} facets at the truncated edges and corners. These tiny {111} facets can reconstruct in N_2_ plasma, but not in air plasma, because N_2_ plasma has a larger reshaping capability for the {111} facets than air plasma. The reconstruction on the tiny {111} facets together with the heating leads to the slight shape change for the NRs in the N_2_ plasma treatment.

**Figure 5:**
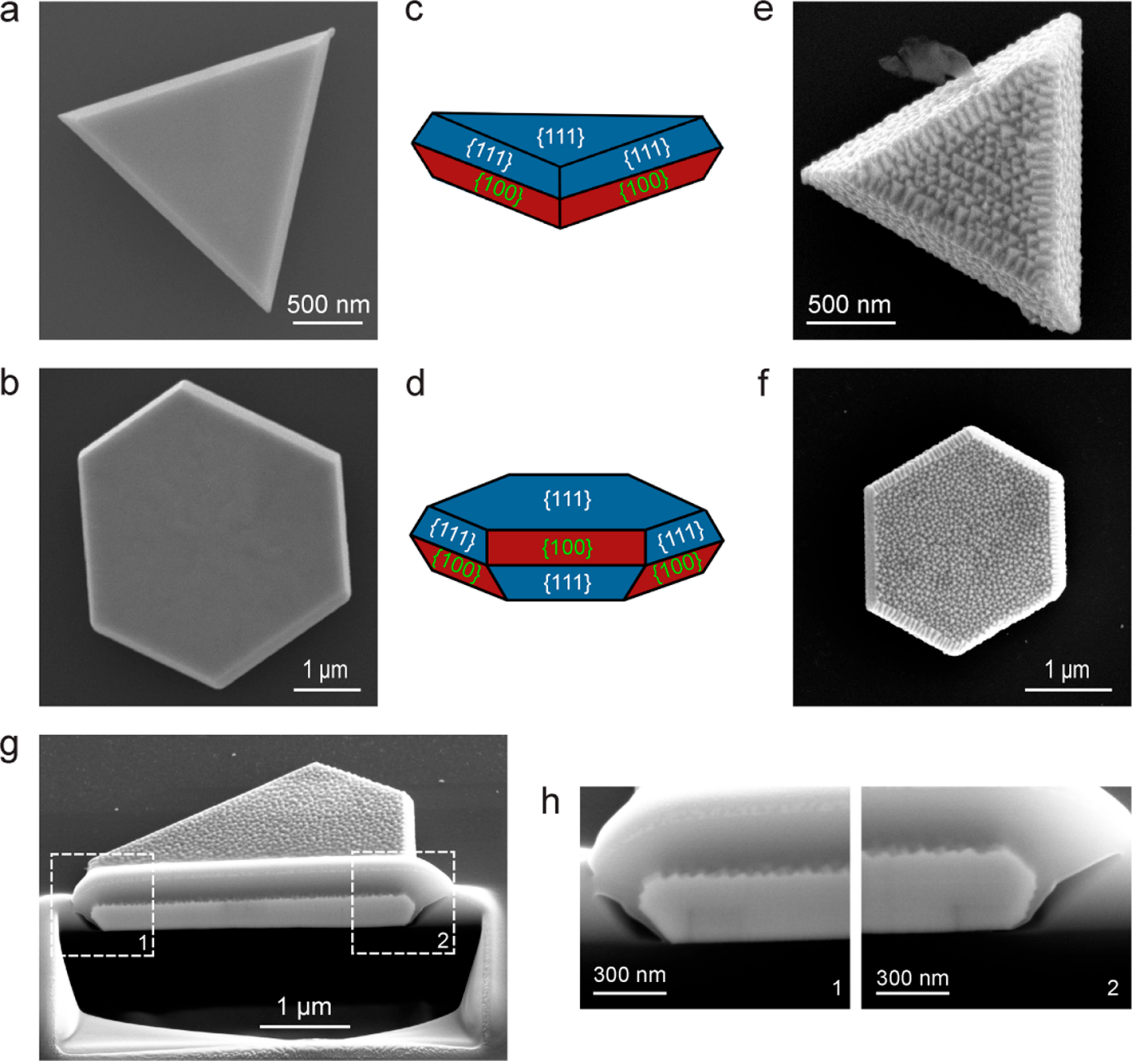
Large Au NPLs before and after the N_2_ plasma treatment. (a,b) SEM images of a large triangular and a hexagonal Au NPL before the plasma treatment, respectively. (c,d) Schematics of the large triangular and hexagonal Au NPLs, respectively, showing the crystalline facets of the top and side surfaces. (e,f) SEM images of a large triangular and a hexagonal Au NPL after the N_2_ plasma treatment, respectively. (g) Low-magnification cross-sectional SEM image of a N_2_ plasma-treated, FIB-cut, large hexagonal Au NPL. (h) High-magnification cross-sectional SEM images of the two boxed regions in (g).

To further verify the facet-dependent reshaping by the plasma treatment, large Au NPLs of a few micrometers in the edge length were synthesized and treated in the different gaseous environments. The large Au NPLs can be triangular or hexagonal (Figure 5a,b). Such large platelike structures, including both Au and Ag, have been shown to consist of the {111} facets on the top and bottom. There are twinning planes aligned parallel to the top and bottom surfaces in the interior of the NPLs.^37,44−46^ The side facets of the large Au NPLs depend on the number of the twinning planes and the overall geometrical shape. In our experiments, there are only two side facets at any edge, no matter whether the NPL is triangular or hexagonal, meaning that there is likely only one twinning plane in each large NPL. The side surfaces of the large triangular Au NPLs are composed of the {111} and {100} facets at each edge (Figure 5c). The stacking order is the same at all of the three edges.^45,50,51^ The side surfaces of the large hexagonal NPLs alternate both vertically and laterally between the {111} and {100} facets (Figure 5d), as can be modeled by a twinned Wulff construction.^37,47,48^ The {111} facets of the large Au NPLs are reshaped into right pyramids after the N_2_ plasma treatment, while the {100} facets remain unchanged (Figure 5e,f). To further observe the reshaping behavior on the side facets of the large Au NPLs, a large hexagonal Au NPL treated by N_2_ plasma was cut with FIB. Before FIB cutting, a thin layer of Pt was deposited to protect the Au NPL from the damage caused by the high-energy ion beam. The cross-sectional SEM images (Figure 5g,h) reveal that only the {111} facets are reshaped, while the {100} facets remain unchanged after the N_2_ plasma treatment. The same results were observed in the air plasma treatment for both large triangular and hexagonal Au NPLs on the top SEM view (Figure S15) and for the FIB cutting of a large hexagonal Au NPL on the cross-sectional SEM view (Figure S16). In contrast, the large Au NPLs cannot be reshaped in the O_2_ or Ar plasma treatment (Figure S17). Only the sharp vertexes of the large triangular Au NPLs are slightly changed after the O_2_ and Ar plasma treatment, which can be ascribed to the plasma-induced heating. Taken together, these results show clearly that the air and N_2_ plasma treatment only causes the reshaping on the {111} facets of the Au NPLs. On the other hand, because the plasma treatment can only cause reconstruction on the {111} facets and the {100} facets are stable, the produced pyramids on the reconstructed surfaces should be right pyramids. The three exposed surfaces of each pyramid should be the {100} facets.

**Figure 6:**
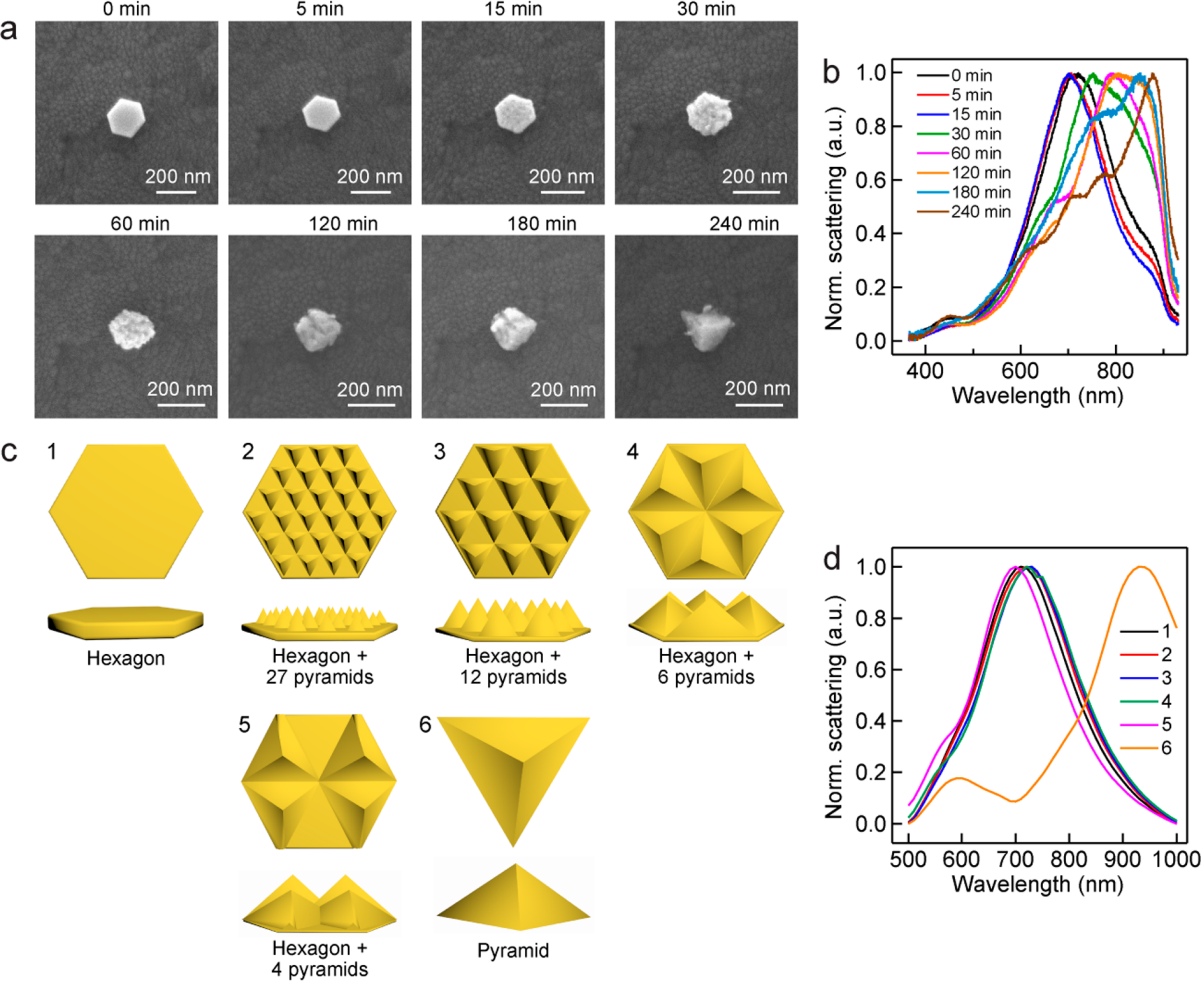
Evolutions of the morphology and plasmon resonance of a gold NPL treated in N_2_. (a) SEM images of the Au NPL treated for increasing cumulative periods of time. The NPL is from the 41 nm thick Au NPL sample. (b) Normalized scattering spectra of the treated Au NPL shown in (a). The indicated treatment durations are cumulative. (c) Schematics showing the models of the reshaped Au NPL used for the DDA simulations. (d) Scattering spectra simulated according to the models shown in (c).

The plasmon resonance of the plasma-treated Au NPLs was further investigated by single-particle dark-field scattering measurements. The 41 nm thick Au NPL samples were deposited on indium–tin oxide (ITO)-coated glass substrates, because the transparency and electrical conductivity of ITO substrates allow for both SEM imaging and dark-field scattering measurements. The same NPLs were located under both SEM and dark-field scattering imaging after each step of the plasma treatment. The shape of the individual Au NPLs is gradually changed to a pyramid after being treated for a total of 240 min in N_2_ plasma (Figure 6a). The Au NPLs were synthesized with CTAB surfactant in aqueous solutions. They are capped with CTAB molecules. No significant changes are observed on the surfaces of the NPLs in the first 5 min, during which the protecting CTAB molecules are believed to be removed by the plasma treatment and small right pyramids start to form. When the treatment time is increased to 15 min, the pyramids grow larger. With the increase in the treatment duration, the number of the right pyramids decreases, the pyramids grow larger, and the NPL becomes thinner (Figure 6a). A large right pyramid is finally formed. The plasmon resonance peak of the same Au NPL exhibits slight blueshifts in the first 15 min of the plasma treatment due to the removal of the CTAB molecules and the slight surface reconstruction (Figure 6b). As the cumulative treatment time is further increased, the plasmon resonance peak shows large redshifts, which can be ascribed to the gradual reconstruction from the plate shape to a single right pyramid. The integrated scattering intensities exhibit fluctuations in the beginning and then decrease at longer plasma treatment time (Figure S18). The fluctuations are caused by the changes in the focus during the scattering measurements, and they later drop in intensity is because the plasmon peak is shifted out of the optical detection range. To better understand the evolution of the plasmon peak with the shape changes of the plasma-treated Au NPLs, optical scattering simulations were performed in the discrete dipole approximation (DDA).^49^ As illustrated schematically in Figure 6c, six models were considered for the DDA simulations with all maintaining the same volume. These include the original hexagonal Au NPL, the final single right pyramid, and four intermediate structures, which are composed of a hexagonal NPL and a number of supported right pyramids. As the size of the right pyramids increases, their number is reduced from 27 to 4 while the hexagonal NPL thickness is decreased to allow for a constant volume. The simulated plasmon peaks for models 1–5 are located spectrally very close to each other while that for model 6 shows a large redshift (Figure 6d). The closeness among the plasmon peaks of models 1–5 is believed to result from the overall hexagonal shape. The discrepancy between the measured and simulated scattering spectra suggests that the overall shape of the NPL starts gradually deviating from the hexagonal shape after 15 min of the plasma treatment.

Dark-field scattering measurements were also performed on a gold NPL plasma treated in air for different cumulative periods of time (Figure S19). Similar to the case with the N_2_ plasma treatment, the formed right pyramids increase in size and decrease in number, and the overall shape of the nanoparticle changes from hexagonal to triangular. The plasmon resonance peak only shows small shifts in comparison with its original spectral position, even for the one plasma-treated in air for the longest time, and the spectral shape remains nearly the same. The integrated scattering intensities exhibit small fluctuations without clear reduction (Figure S20). These results suggest that the evolutions of the morphology and plasmon resonance are dependent on the gaseous environment during the plasma treatment. To further verify the inability of O_2_ plasma to reshape the Au NPLs, dark-field scattering measurements were also performed on the 41 nm thick Au NPLs before and after the O_2_ plasma treatment (Figure S21). The Au NPL remains unchanged in morphology after the O_2_ plasma treatment. The plasmon resonance peak only shows a slight blueshift due to the removal of the adsorbed CTAB molecules by O_2_ plasma. Raman measurements were also performed on the 41 nm thick Au NPL sample treated in N_2_, air and O_2_ for different periods of time to investigate the CTAB molecules adsorbed on the Au NPLs (Figure S22). The vibration peaks ascribed to the CTAB molecules at 758 and 1448 cm^–1^ disappear after the plasma treatment in N_2_, air, and O_2_ for 5, 1, and 1 min, respectively. The disappearance of the Raman peaks suggests that the CTAB molecules on the surface of the Au NPLs are destroyed by the plasma treatment in a few minutes.

We finally performed refractive index sensitivity (RIS) and SERS measurements on the plasma-treated Au NPLs and compared the performances between the plasma-treated and untreated ones. The RIS is defined as the plasmon shift in wavelength as the surrounding dielectric medium is increased by one refractive index unit (RIU). It has been widely employed to evaluate the sensitivities of plasmonic nanoparticles with different sizes and shapes to the index changes in the surrounding medium. For the RIS measurements, the 19 nm thick Au NPLs were selected. They were adsorbed on glass substrates because glass substrates can adsorb a sufficient amount of CTAB-capped Au nanocrystals for ensemble extinction measurements.^34^ The glass substrates with the adsorbed Au NPLs were immersed in water–glycerol mixtures of different compositions for the extinction measurements. The refractive index of the solvent mixture was changed by adjusting the composition. The plasmon peak of the Au NPLs immobilized on glass substrates redshifts as the refractive index of the solvent mixture is increased. The dependence of the dipole plasmon resonance wavelength on the refractive index of the solvent mixture can be linearly fitted. The slope gives the RIS. The RIS of the 19 nm thick Au NPLs without plasma treatment was determined to be 330 ± 20 nm/RIU (Figure S23). Because the Au NPLs in our study are supported on substrates, their RISs are generally smaller than those measured when they are dispersed in solutions.^18^ The same Au NPLs plasma-treated in air for 30 and 60 min exhibit the RISs of 350 ± 2 nm/RIU and 357 ± 15 nm/RIU, respectively (Figures S24 and S25). The plasma treatment causes a slight increase in RIS for the Au NPLs. The increase is believed to arise from two factors that are associated with the generated right pyramids. The right pyramids increase the surface area and hence the effective sensing volume. The sharp vertexes and edges of the right pyramids can bring large local electric field enhancement. Both the increased sensing volume and the enlarged field enhancement can cause higher RISs.^36^

**Figure 7:**
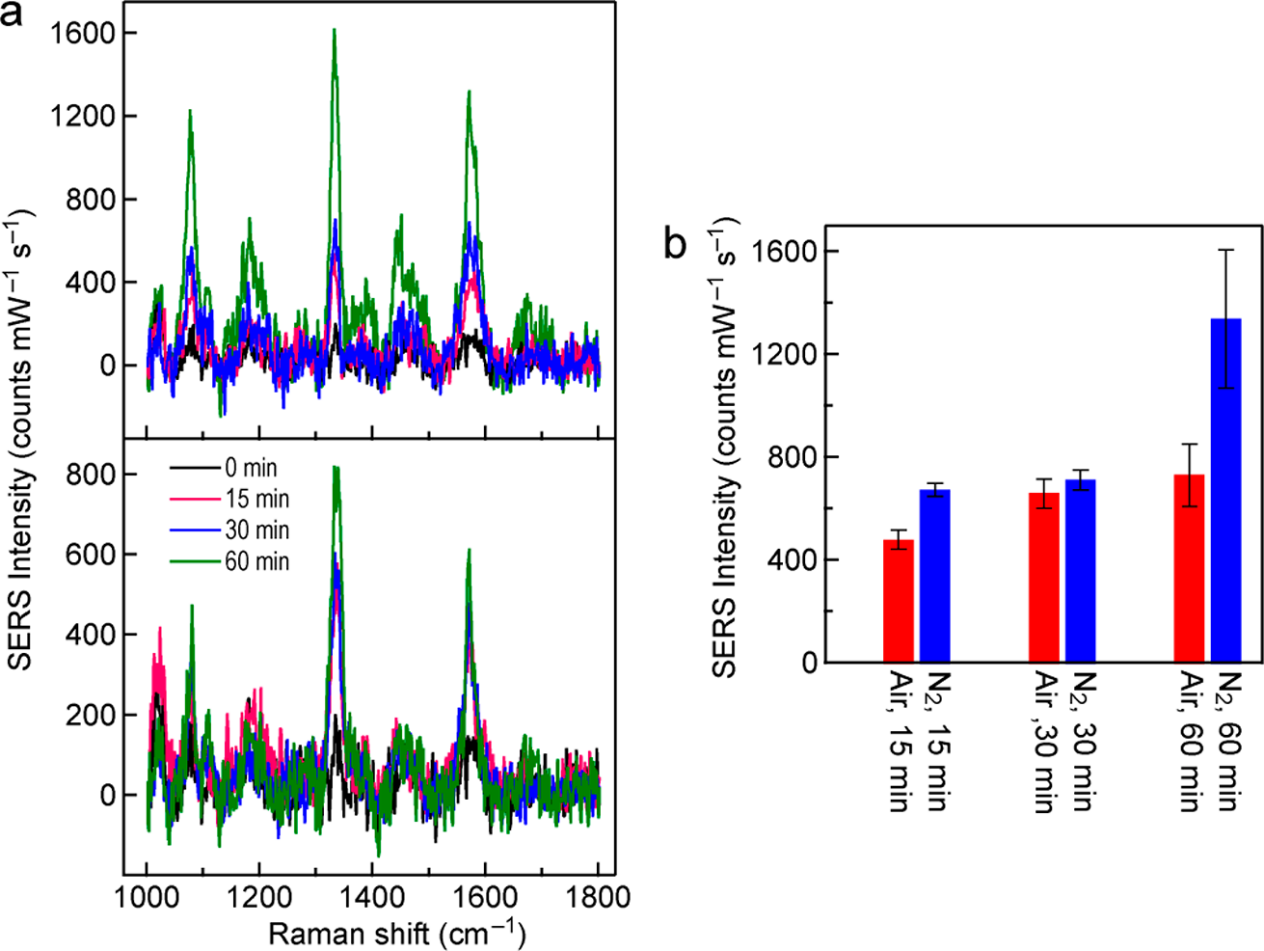
SERS measurements on the plasma-treated, 53 nm thick Au NPLs. (a) Representative SERS spectra of the Au NPLs treated in N_2_ (top) and air (bottom) for increasing periods of time. The optical power of the excitation laser at 633 nm is 0.225 mW, and the integration time is 10 s. (b) Average integral SERS intensities of the dominant Raman peak at 1334 cm^–1^ for the Au NPLs treated under the different conditions.

The SERS measurements were performed on the 53 nm thick Au NPLs because of their large scattering capability. The Au NPLs were deposited on ITO substrates and plasma-treated in N_2_ and air for different periods of time (Figures S26 and S27). The plasmon resonance peak of the untreated Au NPLs was located at ∼700 nm. 4-nitrothiophenol (4-NTP) was chosen as the probe molecule, which can attach on the surface of the Au NPLs through the formation of the Au–S bond.^52^ Under our measurement conditions, the Raman signals of two or three well-separated Au NPLs were typically collected in each measurement. Figure 7a shows the representative Raman spectra measured on the Au NPLs treated in N_2_ and air for 0, 15, 30, and 60 min, respectively. In all spectra, the prominent peak around 1334 cm^–1^ originated from O–N–O stretching.^53^ The Raman peaks at 1076 and 1571 cm^–1^ come from C–S stretching and the phenyl ring, respectively.^53^ To better compare the SERS intensities, the intensity of the dominant Raman peak at 1334 cm^–1^ (Raman scattering wavelength, 750 nm) of 4-NTP molecules was averaged from 10 measurements at different positions on the ITO substrate for each case and normalized against the excitation laser power and exposure time (Figure 7b). No clear 4-NTP Raman signals were detected from the Au NPLs without plasma treatment. Their average intensities were omitted in Figure 7b. The Raman intensities on the N_2_ plasma-treated Au NPLs are higher than those on the air plasma-treated ones. After the N_2_ plasma treatment, the Raman intensity of the Au NPLs treated for 60 min is ∼2-fold larger than that of the ones treated for 15 and 30 min, and the intensities of the Au NPLs treated for 15 and 30 min are nearly equal. After the air plasma treatment, the Raman intensities only increase slightly with the treatment time. After the plasma treatment, the surfaces of the Au NPLs become rough. As a result, their surface areas become larger, and there are more hotspots on the surfaces. The increased areas can adsorb more probe molecules. These factors cause the increases in the Raman signal. In addition, the maximal SERS enhancement factor has been shown to occur for the plasmon wavelength to be adjusted in between the excitation laser wavelength and Raman emission wavelength.^54^ In our experiments, the plasmon resonance peak of the air plasma-treated Au NPLs remains nearly unchanged at 700 nm (Figure S27). The plasmon peak of the N_2_ plasma-treated Au NPLs redshifts considerably (Figure S26). Since the excitation laser wavelength in our experiments is 633 nm, the increases in the Raman signal can be mainly attributed to the increases in the surface area of the Au NPLs after the plasma treatment.

## Conclusion

We have demonstrated that the plasma generated in a common lab plasma cleaner can reshape Au NPLs deposited on different substrates. After the plasma treatment, the {111} surfaces of the Au NPLs become rough, leading to the formation of right pyramids with {100} facets exposed. When the treatment time is increased, the Au NPLs become thinner, the right pyramids gradually become larger, and their number decreases. The reshaping process is gas-dependent. It only occurs in the gaseous environment that contains nitrogen. Moreover, the reshaping process is also facet-dependent. It can only be induced on the {111} facets of Au. With the increase of the plasma treatment extent, the morphology of the Au NPLs changes from the original hexagonal plate, through the intermediate stages where the number and size of the formed right pyramids gradually evolve, to the final single right pyramid. The morphology evolution is accompanied by plasmon redshifts. The reshaped Au NPLs with formed right pyramids possess slightly higher RISs and largely increased SERS signals. Our findings provide a simple means for reshaping noble metal nanocrystals. They will also inspire further studies on the fundamental interactions between the ionic species in plasma and the specific facets of noble metal nanocrystals, which can lead to the development of approaches for modifying the surfaces of noble metal nanocrystals for applications in catalysis and nanoplasmonics.

## Experimental Section

### Preparation of the Hexagonal Au NPLs

The hexagonal Au NPL samples were synthesized using a previously described seed-mediated method.^36^ Five Au NPL samples with different thicknesses were obtained by performing overgrowth on a rounded triangular Au NPL sample with increasing amounts of HAuCl_4_. Each overgrowth solution was prepared by mixing water, CTAB (0.1 M, 0.5 mL), HAuCl_4_ (0.1 M), and ascorbic acid (0.1 M) in sequence. The added volumes of the HAuCl_4_ solution were 40, 80, 120, 160, and 200 μL, respectively. For each overgrowth solution, the volume of the ascorbic acid solution was a half of the HAuCl_4_ solution, and water was added to adjust the total volume to 4 mL. To initiate overgrowth, 1 mL of the rounded triangular Au NPL solution with the extinction value at the major plasmon peak adjusted to 3.0 in advance with water was added into each overgrowth solution. The resultant mixture solution was kept in an isothermal oven at 30 °C for 10 h to complete the growth of the hexagonal Au NPLs.

### Plasma Treatment of the Au NPLs

Si, glass, or ITO substrates were first ultrasonicated in ethanol for 1 h, followed by plasma treatment for 5 min in an air environment. The as-prepared Au NPLs were rinsed twice and diluted in water. The CTAB-capped NPLs were then deposited on the cleaned substrates at appropriate surface number densities. The Au NPLs on the substrates were treated in a plasma cleaner (Harrick Scientific, PDC-32G, 18 W) for different periods of time in air, N_2_, O_2_, and Ar gas environments.

### Electron Tomography

The HAADF STEM images of the Au NPLs deposited on Si_3_N_4_ membranes were acquired on a Titan Krios operated at 200 kV from −70° to +70° with 1° steps from +60 to +70° and −60 to −70°, and 2° steps from −60 to +60°. The tilt series were registered using a phase-correlation algorithm, and the tilt axes were subsequently adjusted manually to minimize streaking in the reconstructions. Tomographic reconstruction was formulated using a compressed sensing regularization algorithm based on a previous method,^56^ and implemented with 200 iterations of a Chambolle-Pock optimization algorithm, which led to a stable solution for each series.^57^

### Numerical Simulations

The optical scattering spectra were obtained numerically in the discrete dipole approximation method using the DDSCAT code.^49^ The frequency-dependent refractive index of metallic Au was taken from Johnson and Christy^58^ and the ambient refractive index was set to 1.2. To simulate the experimental conditions of dark-field scattering, the incident light was orthogonally polarized and set to form an angle of 31° with the hexagon plane. All calculations were carried out with dipole distances of ∼2.5 nm and a wavelength sampling of 10 nm.

### Characterization

Extinction spectra were measured on a PerkinElmer Lambda 950 ultraviolet/visible/near-infrared spectrophotometer using plastic cuvettes of an optical path length of 1.0 cm. SEM imaging was carried out on a JEOL JSM-7800F Schottky field-emission scanning electron microscope operated at 10 kV. AFM images for the Au NPL samples without plasma treatment were acquired in air on a Veeco Metrology system (Model No. 920-006-101) that was operated at the contact mode using a supersharp silicon nitride AFM tip (Bruker). AFM images for the plasma-treated Au NPL samples were taken on the same system, which was operated at the tapping mode. Single-particle dark-field scattering spectra were recorded on an Olympus BX60 upright microscope that consists of a quartz–tungsten–halogen lamp (100 W), a monochromator (Acton, SpectraPro 2360i), and a charge-coupled device camera (Princeton Instruments, Pixis 400, cooled to −70 °C). A 100× dark-field objective (numerical aperture 0.9) was employed for both exciting the individual nanoparticles with the white light and collecting the scattered light. The RIS of the immobilized Au NPLs was measured by a previously reported method.^34^ Before RIS measurements, the Au NPLs with the thickness of 19 nm were deposited on glass substrates. Water–glycerol mixtures with the volume percentage of glycerol varied among 0, 10, 30, 50, 70, and 90% were used to change the refractive index of the surrounding medium. The refractive indexes of the mixtures were calculated according to the Lorentz–Lorenz equation,^55^ where the refractive indexes of water and glycerol are 1.3334 and 1.4746, respectively. The Raman spectra for the CTAB molecules were taken using a hand-held Raman spectrometer (Ocean Optics, ACCUMAN SR-501 Pro system). The excitation wavelength was 785 nm with a laser spot size of ∼0.2 mm and a laser power of 300 mW. The integration time was 10 s. The Raman spectra for the SERS study were acquired on a Renishaw inVia Reflex system. The excitation wavelength was 633 nm with a laser spot size of ∼1 μm and a laser power of 0.225 mW. A 100× objective (numerical aperture 0.9) was employed and the integration time was set at 10 s.

## Supporting Information Available

AFM results of the Au NPL samples; additional SEM images and extinction spectra of the nanocrystals; additional 3D electron tomography results of the reshaped Au NPL samples; additional XPS spectra of the Au NPL samples; additional scattering spectra of the Au NPL samples; RIS measurement results of the Au NPL samples (PDF)
