# Supplementary material for: Facet- and Gas-Dependent Reshaping of Au Nanoplates by Plasma Treatment
Source: ACS Nano. 2021 Jun 11;15(6):9860–70. doi: 10.1021/acsnano.1c00861 (PMC8223482; doi:10.1021/acsnano.1c00861)
Supplement: Supplementary file 1 — nn1c00861_si_001.pdf [file nn1c00861_si_001.pdf]

Supporting Information

Facet- and Gas-Dependent Reshaping of Au Nanoplates by Plasma Treatment

Ruoqi Ai,[†] Christina Boukouvala,[‡,§] George Lewis,[‡,§] Hao Wang,[#] Han Zhang,[†] Yunhe Lai,[†] He Huang,[†] Emilie Ringe,[‡,§] Lei Shao,[#] and Jianfang Wang*,[†]*

[†]Department of Physics, The Chinese University of Hong Kong, Shatin, Hong Kong SAR, China

[‡]Department of Materials Science and Metallurgy, University of Cambridge, Cambridge CB3 0FS, United Kingdom

[§]Department of Earth Sciences, University of Cambridge, Cambridge CB2 3EQ, United Kingdom

[#]Shenzhen JL Computational Science and Applied Research Institute, Shenzhen 518109, China

*Corresponding authors. Email: jfwang@phy.cuhk.edu.hk; er407@cam.ac.uk

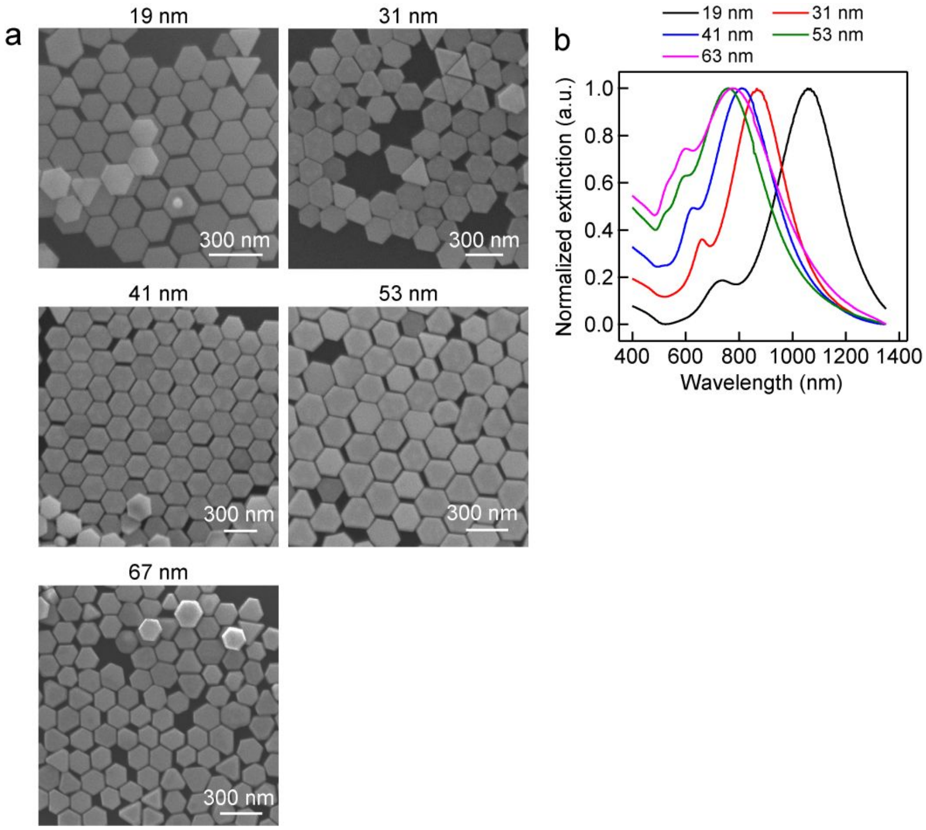

Figure S1. Five Au NPL samples of different thicknesses. (a) SEM images of the samples. The lateral sizes of the Au NPL samples are all ~170 nm. (b) Extinction spectra of the Au NPL samples dispersed in aqueous solutions.

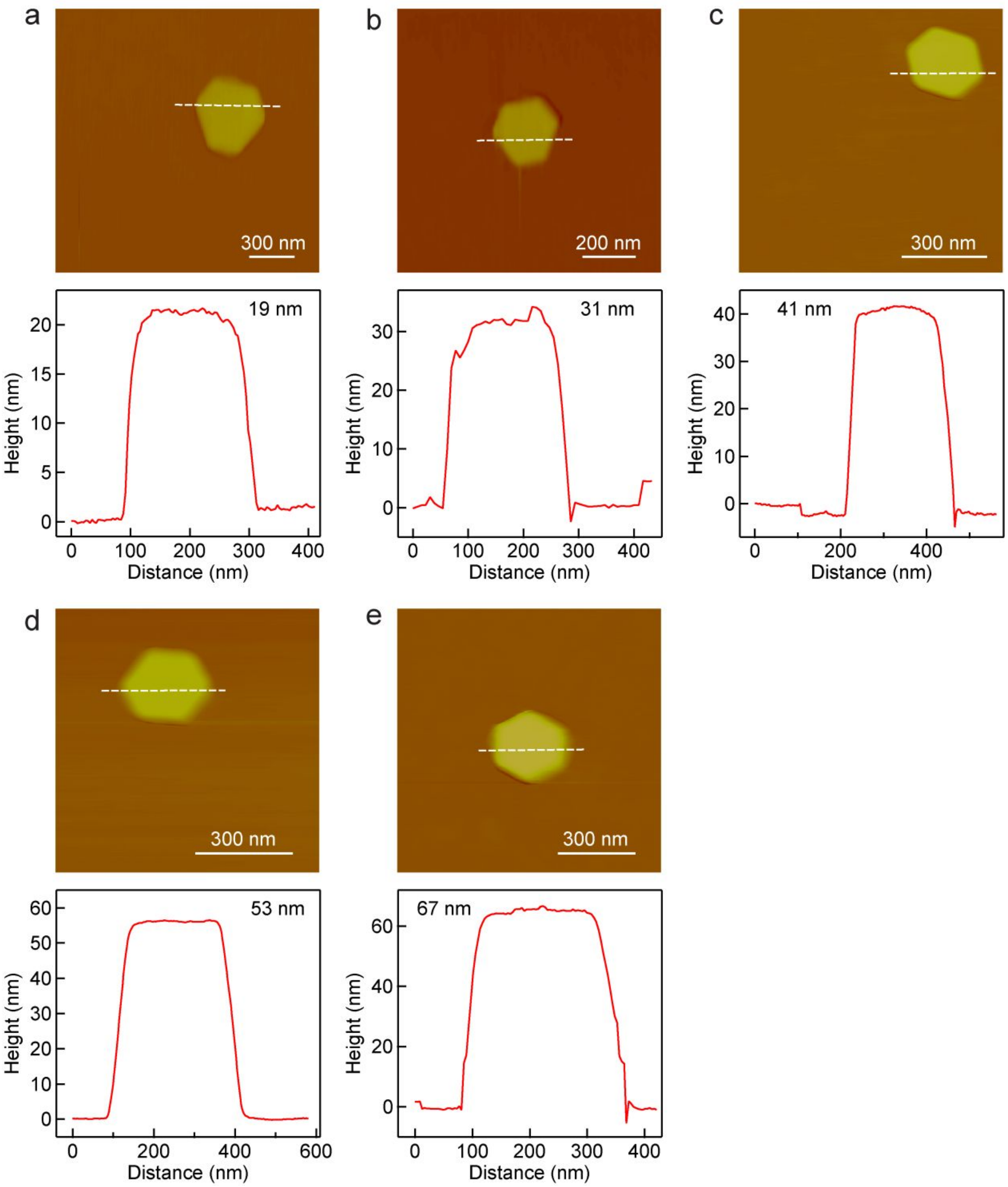

Figure S2. AFM measurements of the Au NPLs. (a–e) AFM height images (first and third rows) and height profiles (second and fourth rows) of the Au NPL samples. The height profiles were extracted along the white dashed lines indicated on the corresponding height images. The average thicknesses of the Au NPL samples are 19 ± 2 nm, 31 ± 1 nm, 41 ± 2 nm, 53 ± 4 nm and 67 ± 5 nm, respectively.

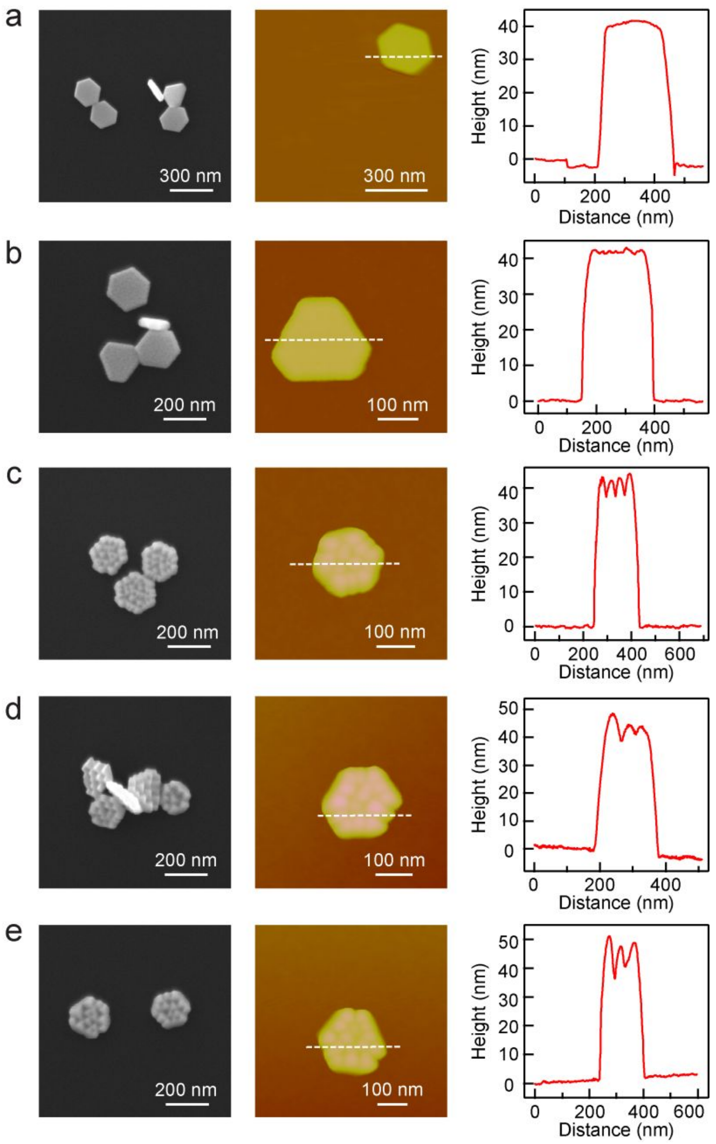

Figure S3. AFM measurements of the air plasma-treated, 41 nm thick Au NPLs. In the left, middle and right columns are the SEM images, AFM height images, and the height profiles extracted along the dashed lines indicated in the AFM height images, respectively. (a–e) Au NPLs plasma-treated for 0, 5, 15, 30 and 60 min, respectively.

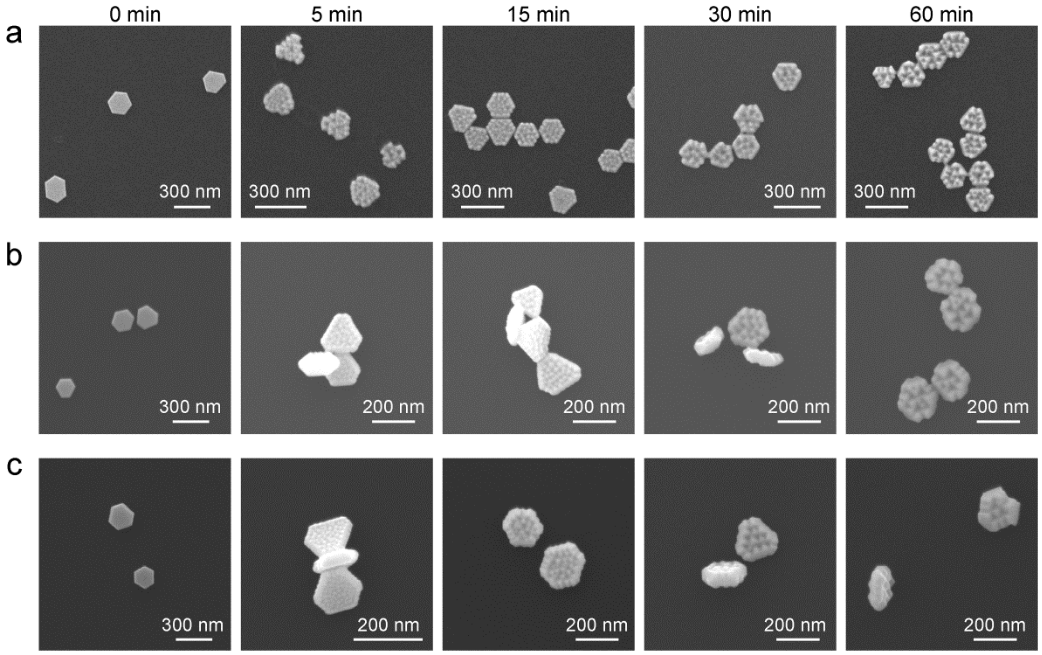

Figure S4. Air plasma treatment of the Au NPLs with different thicknesses. (a) SEM images of the Au NPL sample with the thickness of 31 nm after the plasma treatment in air for 0, 5, 15, 30 and 60 min, respectively. (b) SEM images of the Au NPL sample with the thickness of 53 nm after the plasma treatment in air for 0, 5, 15, 30 and 60 min, respectively. (c) SEM images of the Au NPL sample with the thickness of 67 nm after the plasma treatment in air at 0, 5, 15, 30 and 60 min, respectively.

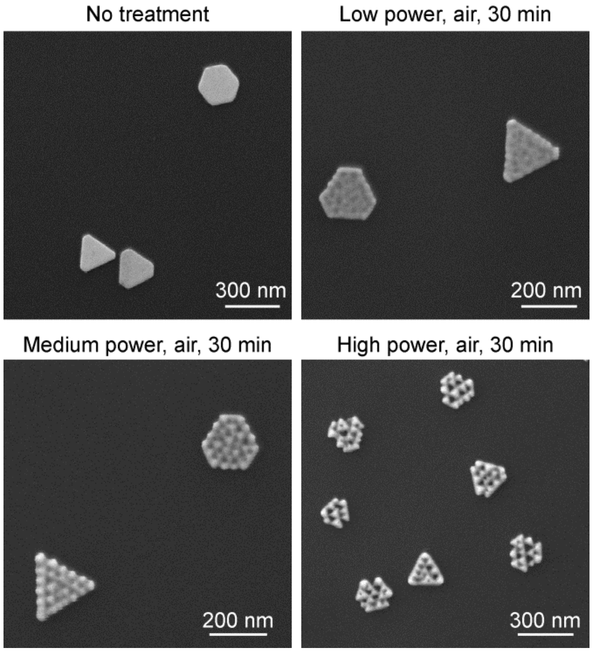

Figure S5. Au NPLs subjected to the air plasma treatment at different powers. Shown are the SEM images of the 19 nm thick Au NPLs that are untreated, treated at the low, medium and high power, respectively.

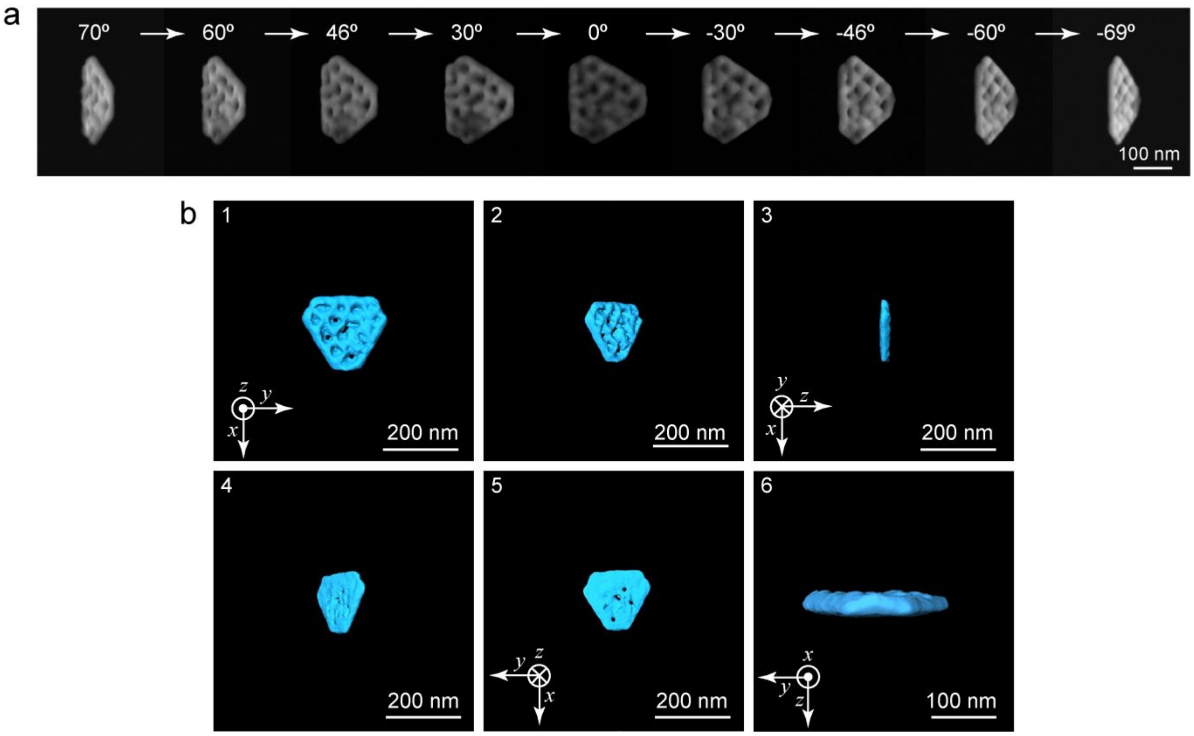

Figure S6. Electron tomography of a reshaped, 19 nm thick Au NPL. (a) HAADF STEM images across the tilt series. The tilt axis is vertical in the page. (b) Reconstructed images of the NPL in

different orientations. The NPL is rotated around the *x*-axis clockwise in the images from 1 to 5. The Au NPL was treated in air plasma for 30 min.

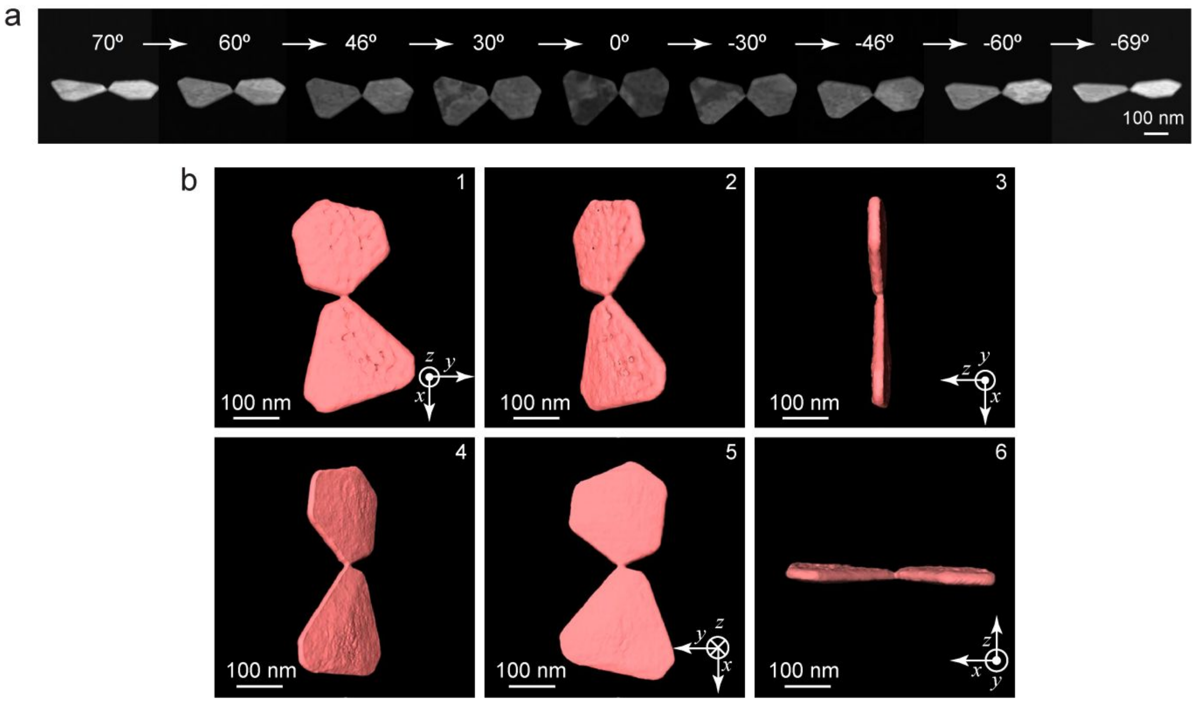

Figure S7. Electron tomography of two reshaped, 19 nm thick Au NPLs. (a) HAADF STEM images across the tilt series. The tilt axis is vertical in the page. (b) Reconstructed images of the NPLs in different orientations. The NPLs are rotated around the *x*-axis counterclockwise in the images from 1 to 5. The Au NPLs were treated in air plasma for 5 min.

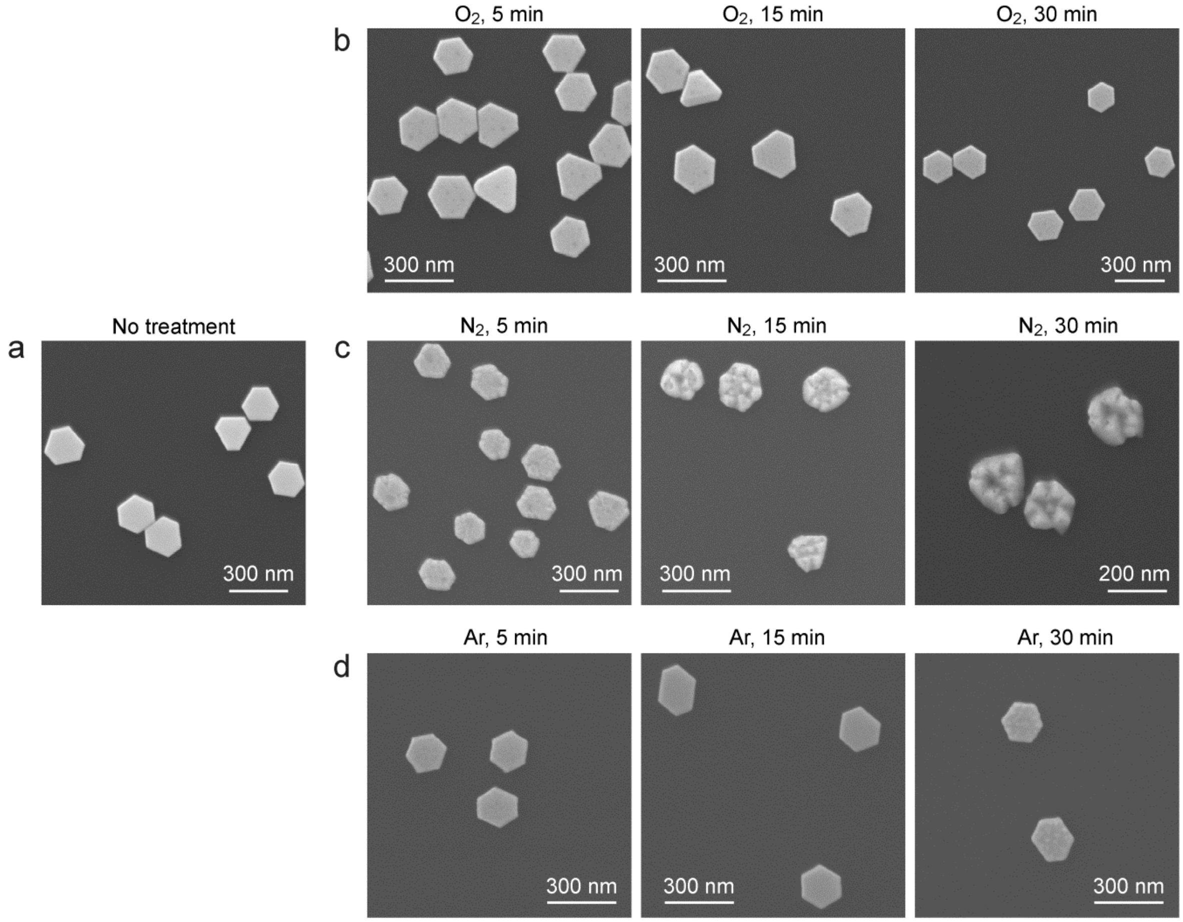

Figure S8. Au NPLs subjected to the plasma treatments in different gaseous environments. (a) SEM image of the 40 nm thick Au NPLs without plasma treatment. (b–d) SEM images of the Au NPLs plasma-treated in O_2, N_2 and Ar, respectively, for different periods of time.

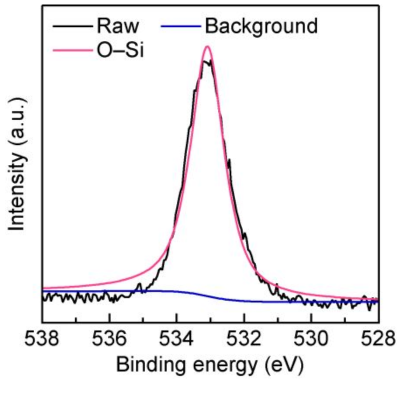

Figure S9. XPS spectrum of the untreated Au NPLs. The peak is for O 1s.

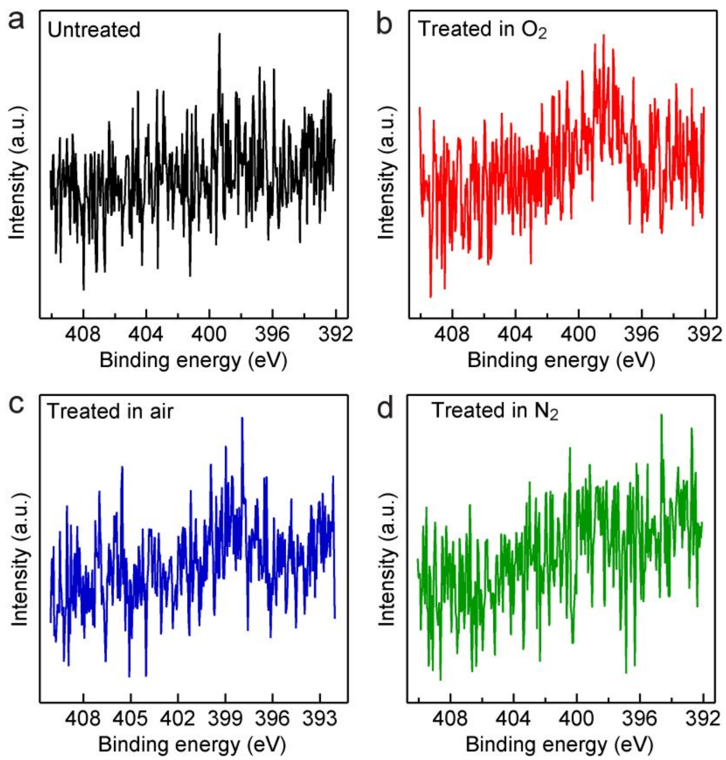

Figure S10. XPS spectra of the 53 nm thick Au NPLs in the N 1s region. (a) Without plasma treatment. (b) After the O_2 plasma treatment. (c) After the air plasma treatment. (d) After the N_2 plasma treatment.

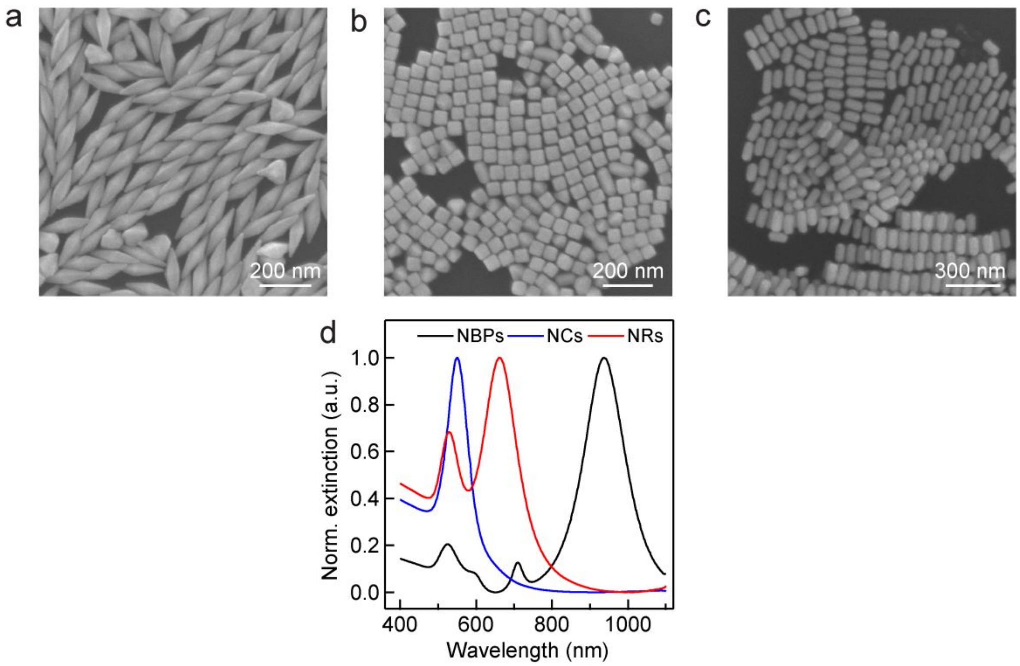

Figure S11. Au nanocrystals with different shapes. (a) SEM image of the Au NBP sample. (b) SEM image of the Au NC sample. (c) SEM image of the Au NR sample. (d) Normalized extinction spectra of the Au NBP, NC and NR samples in aqueous solutions.

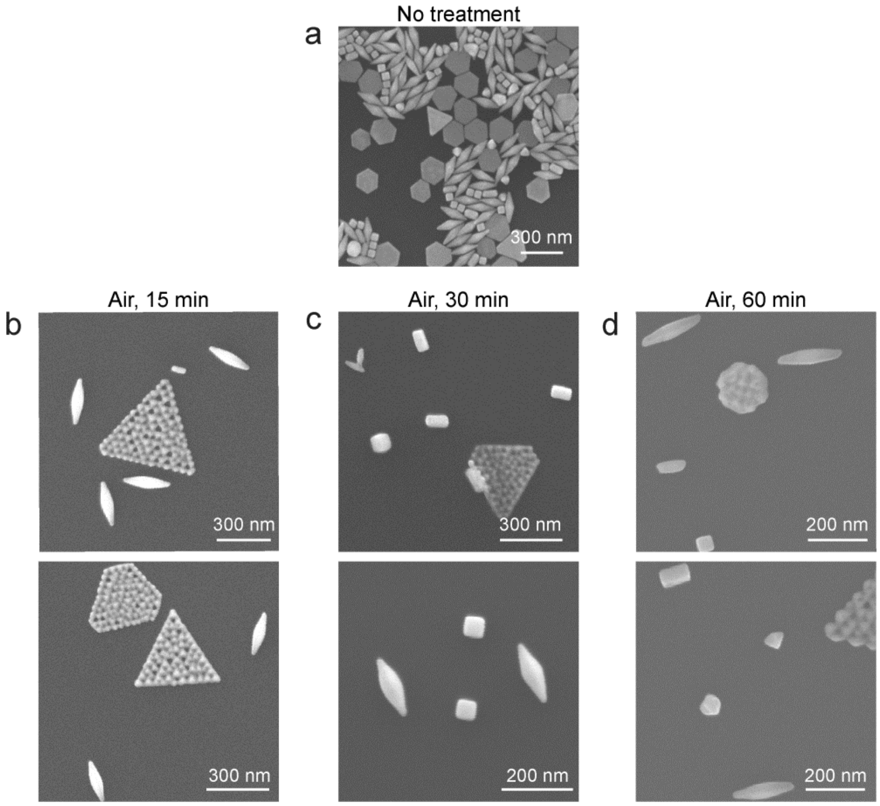

Figure S12. Air plasma treatment of the differently shaped Au nanocrystals. (a) SEM image of the mixed Au nanocrystals before plasma treatment. (b–d) SEM images of the mixed Au nanocrystals treated in air for 15, 30 and 60 min, respectively.

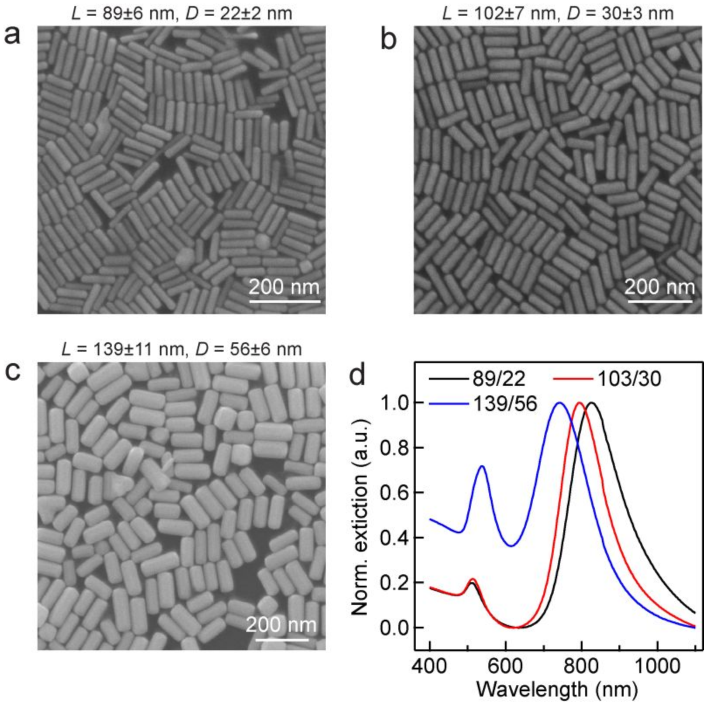

Figure S13. Au NRs with different aspect ratios. (a–c) SEM images of the Au NR samples with average lengths/diameters of 89/22 nm, 102/30 nm and 139/56 nm, respectively. (d) Normalized extinction spectra of the NR samples in aqueous solutions.

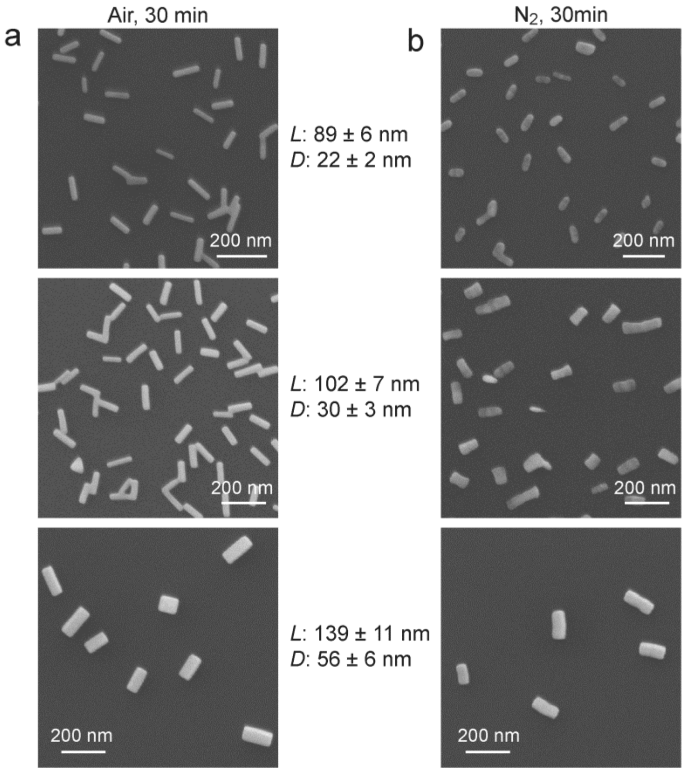

Figure S14. Plasma treatment of the three Au NR samples with different aspect ratios in different gaseous environments. (a,b) SEM images of the Au NR samples treated in air (left) and N_2 (right) plasma for 30 min, respectively.

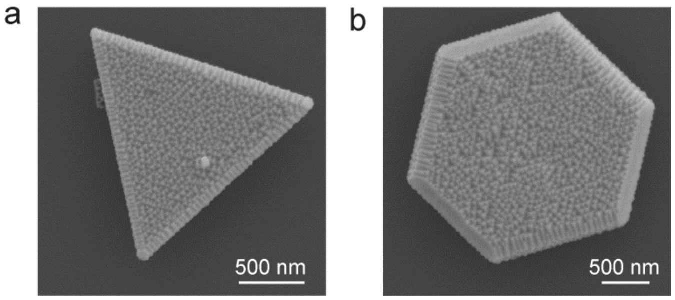

Figure S15. Large Au NPLs after the air plasma treatment. (a) SEM image of a large triangular Au NPL after the air plasma treatment for 30 min. (b) SEM image of a large hexagonal Au NPL after the air plasma treatment for 30 min.

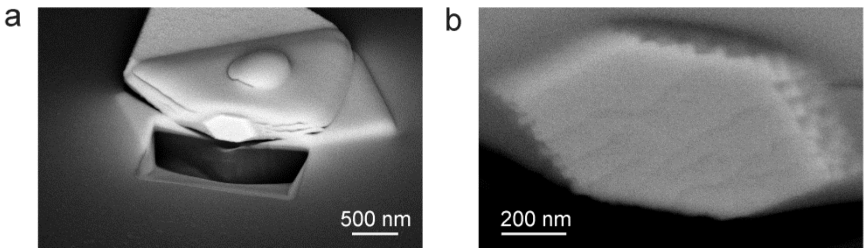

Figure S16. Large Au NPLs after the air plasma treatment for 30 min. (a) Cross-sectional SEM image of an FIB-cut large hexagonal Au NPL at low magnification. The NPL is covered with Pt for protection. Only a small segment at a vertex of the NPL is cut off. (b) Cross-sectional SEM image on the cut NPL at high magnification.

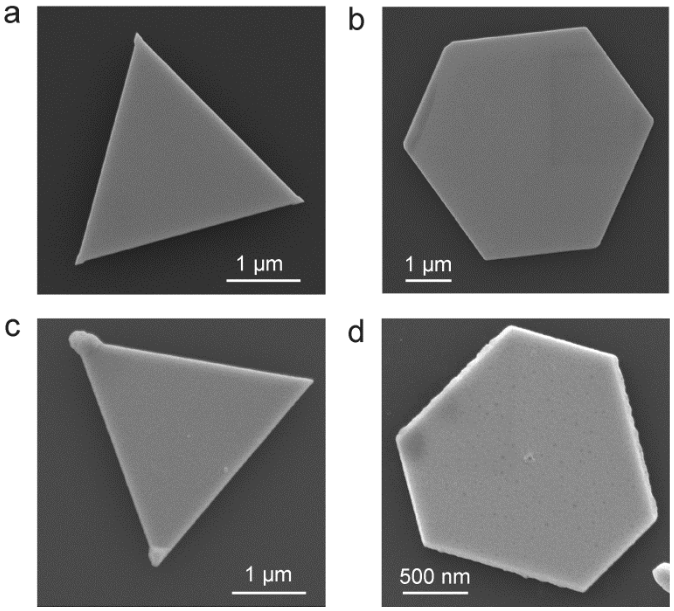

Figure S17. Large Au NPLs after the O_2 and Ar plasma treatment. (a,b) SEM images of a triangular and a hexagonal Au NPL after the O_2 plasma treatment for 30 min, respectively. (c,d) SEM image of a triangular and a hexagonal Au NPL after the Ar plasma treatment for 30 min, respectively.

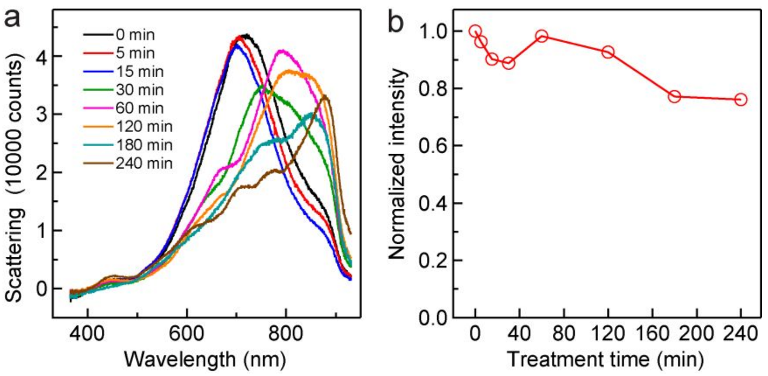

Figure S18. Evolutions of the plasmon resonance peak position and intensity of a gold NPL plasma-treated in N_2. (a) Un-normalized scattering spectra of the treated Au NPL shown in Figure 6a. (b) Integrated scattering intensities plotted as a function of the plasma treatment time. The intensities have been normalized against that of the untreated Au NPL.

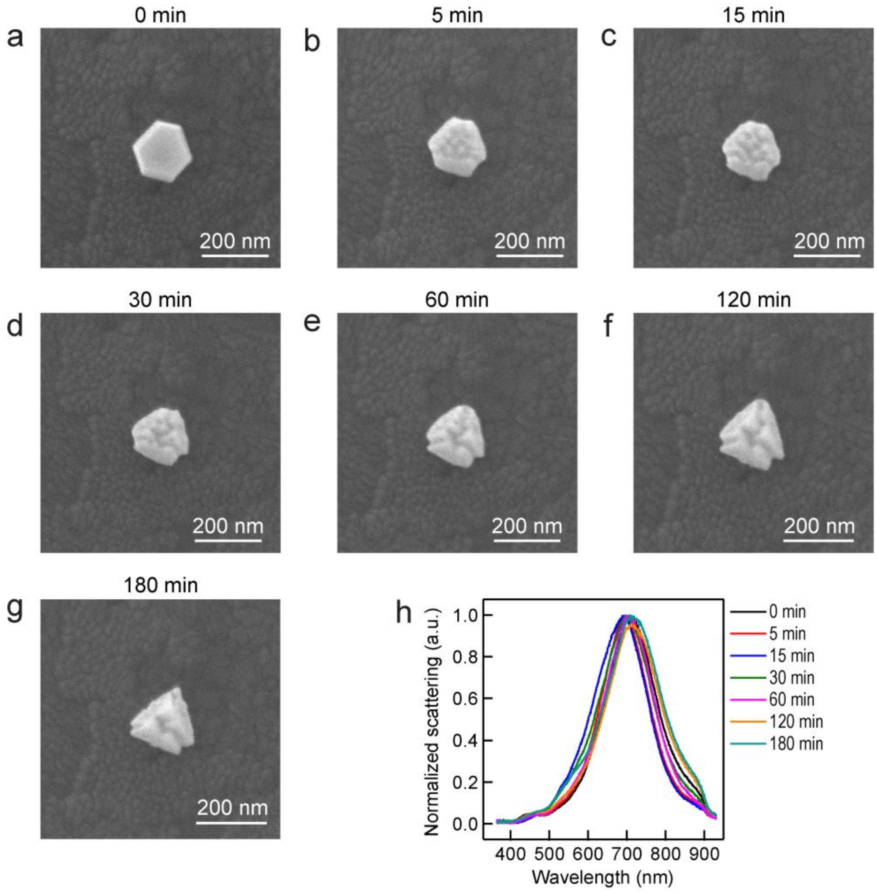

Figure S19. Evolutions of the morphology and plasmon resonance of a gold NPL plasma-treated in air. (a–g) SEM images of the Au NPL plasma-treated for increasing cumulative durations. (h)

Normalized scattering spectra of the treated Au NPL shown in (a−g). The indicated treatment durations are cumulative ones. The NPL is from the 41 nm thick Au NPL sample.

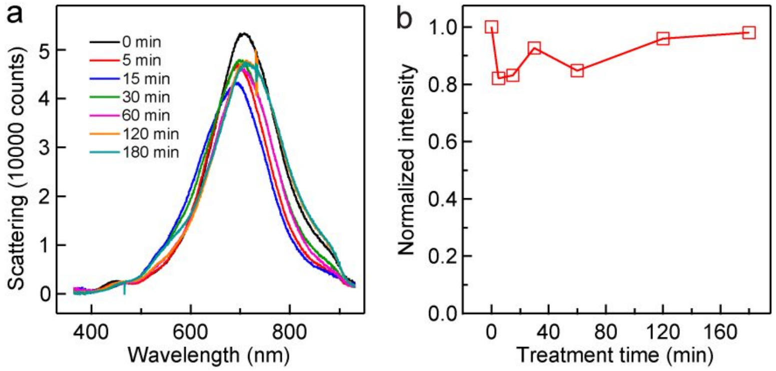

Figure S20. Evolutions of the plasmon resonance peak position and intensity of a gold NPL plasma-treated in air. (a) Un-normalized scattering spectra of the treated Au NPL shown in Figure S19. (b) Integrated scattering intensities plotted as a function of the plasma treatment time. The intensities have been normalized against that of the untreated Au NPL.

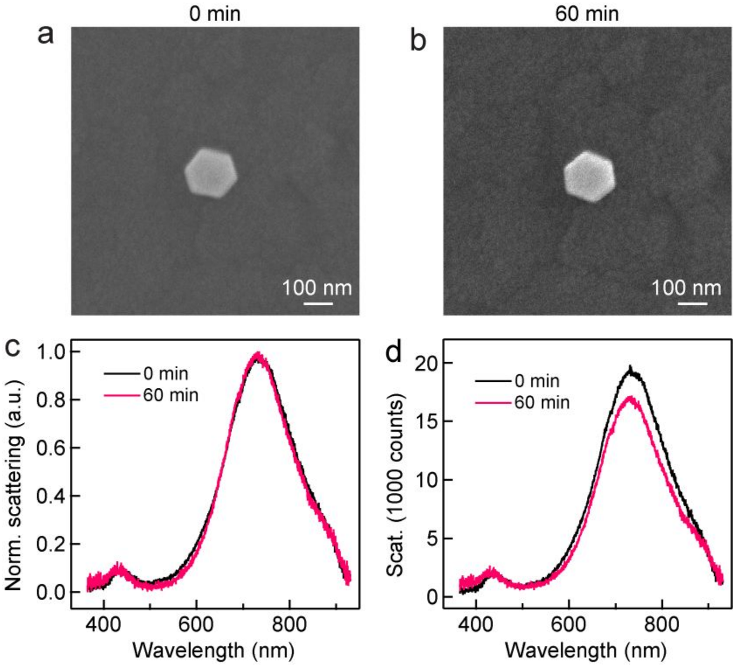

Figure S21. Morphology and plasmon resonance of an O_2 plasma-treated Au NPL. (a,b) SEM images of the Au NPL before and after the O_2 plasma treatment for 60 min. (c) Normalized scattering spectra of the Au NPL before and after the treatment. (d) Un-normalized scattering spectra. The NPL is from the 41 nm thick Au NPL sample.

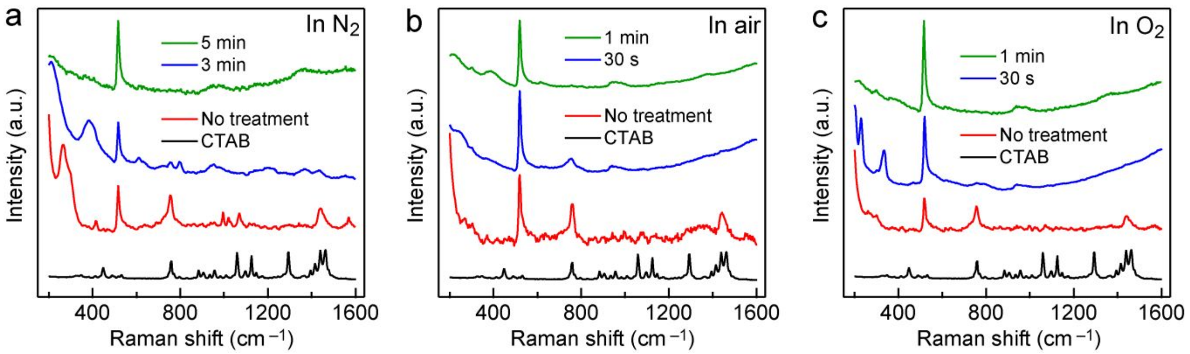

Figure S22. Raman spectra recorded on the 41 nm thick Au NPL sample plasma-treated in the different gas environments. (a) N_2. (b) Air. (c) O_2. The Raman spectra labeled with "CTAB" were measured on pure solid CTAB. The Raman spectra recorded on the NPLs plasma-treated in N_2 for 30 s and 1 min are very similar to that without plasma-treatment. They are not shown in the plot. The major Raman peaks of pure CTAB appear at 758, 1060, 1127, 1296, 1440 and 1464 cm^{-1}. The 758 cm^{-1} peak is attributed to the methyl rocking vibration of the $(CH_3)_3N^+$ group. The peaks at 1060 and 1127 cm^{-1} correspond to the C–C stretching vibrations. The peak at 1296 cm^{-1} is from the CH_2 twisting. The peak at 1440 cm^{-1} is originated from the wag vibration of CH_2. The peak at 1464 cm^{-1} arises from the CH_2 bending vibration. Only the 758 and 1448 cm^{-1} (merged from the two peaks at 1440 and 1464 cm^{-1}) peaks for CTAB molecules are detected on the Au NPL sample without plasma treatment. The other vibration peaks are absent, which can be ascribed to the small amount of the existent CTAB molecules on the Au NPLs. The peak at 520 cm^{-1} comes from the Si substrate. The peaks at 758 and 1448 cm^{-1} for CTAB disappear after the plasma treatment in N_2, air and O_2 for 5, 1 and 1 min, respectively. The absence of the Raman peaks suggests that the CTAB molecules on the surface of the Au NPLs are destroyed by the plasma treatment in a few minutes.

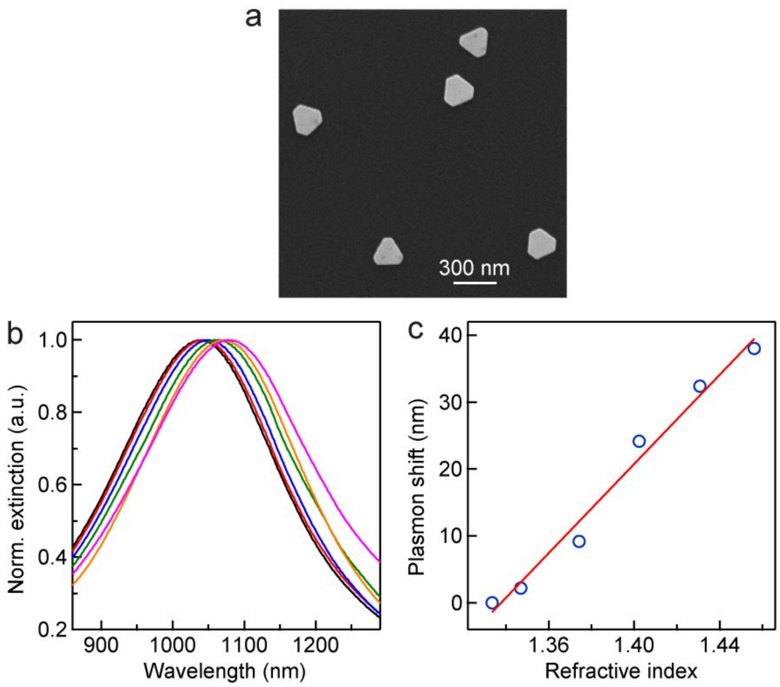

Figure S23. RIS measurement of the 19 nm thick Au NPLs without plasma treatment. (a) SEM image of the Au NPLs adsorbed on a glass substrate. The glass substrate was sputtered with gold to facilitate SEM imaging. (b) Normalized extinction spectra of the Au NPLs immersed in the water–glycerol solvent mixtures of different compositions. (c) Dependence of the dipole plasmon wavelength on the refractive index.

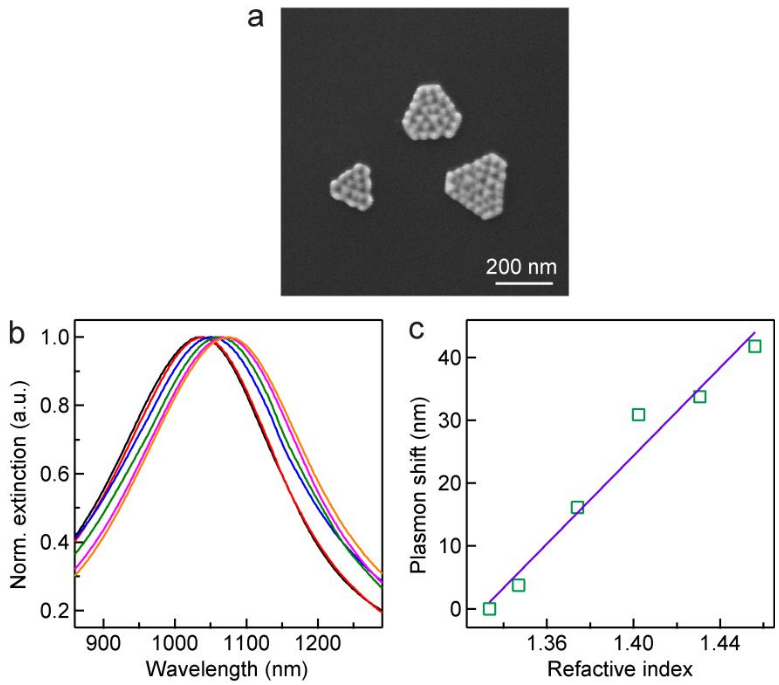

Figure S24. RIS measurement of the 19 nm thick Au NPLs plasma-treated in air for 30 min. (a) SEM image of the plasma-treated Au NPLs. The glass substrate was sputtered with gold to facilitate SEM imaging. (b) Normalized extinction spectra of the Au NPLs immersed in the solvent mixtures. (c) Dependence of the dipole plasmon wavelength on the refractive index.

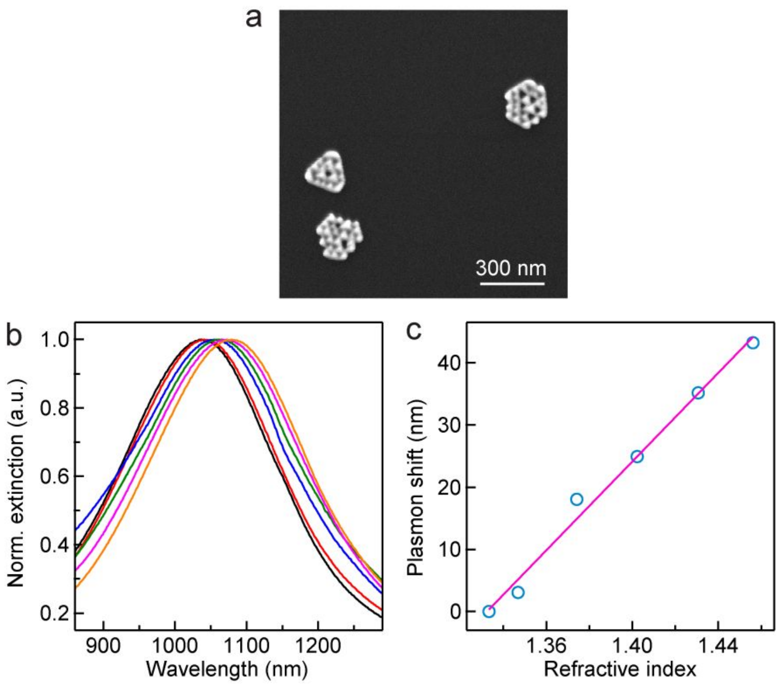

Figure S25. RIS measurement of the 19 nm thick Au NPLs plasma-treated in air for 60 min. (a) SEM image of the plasma-treated Au NPLs. The glass substrate was sputtered with gold to facilitate SEM imaging. (b) Normalized extinction spectra of the Au NPLs immersed in the different solvent mixtures. (c) Dependence of the dipole plasmon wavelength on the refractive index.

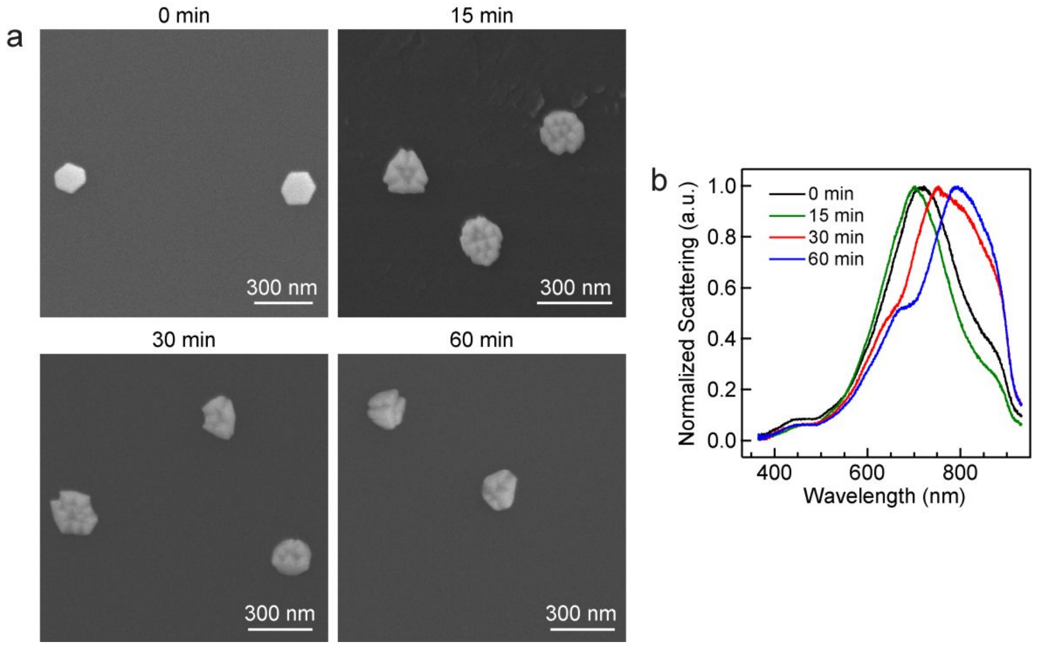

Figure S26. Representative 53 nm thick Au NPLs for the SERS measurements. (a) SEM images of the Au NPLs plasma-treated in N_2 for different periods of time. (b) Normalized scattering spectra of the individual Au NPLs plasma-treated in N_2 for different periods of time.

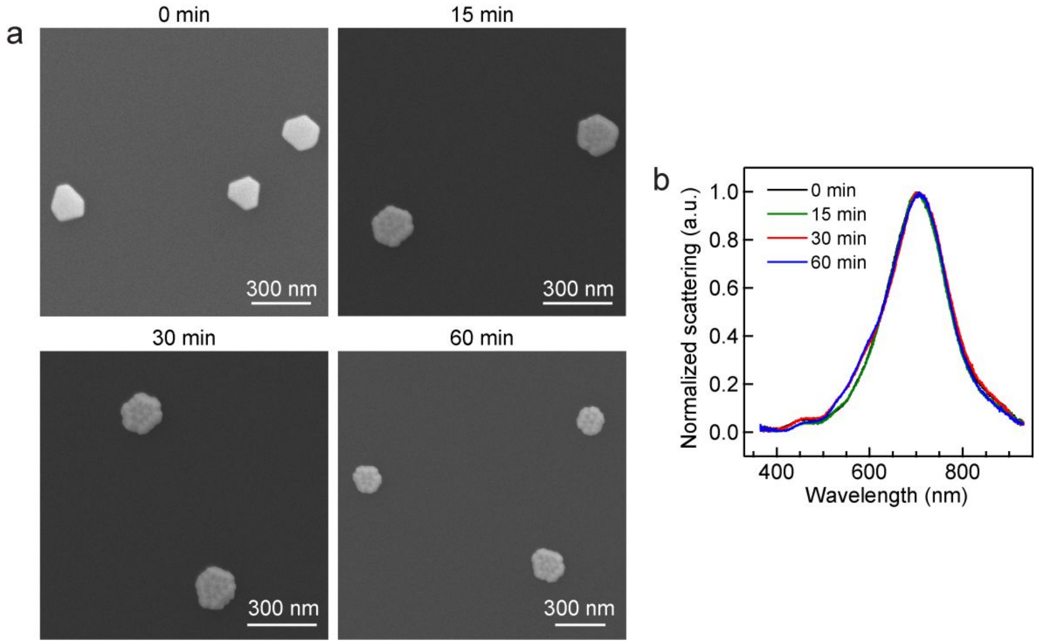

Figure S27. Representative Au NPLs for the SERS measurements. (a) SEM images of the Au NPLs plasma-treated in air for different periods of time. (b) Normalized scattering spectra of the individual Au NPLs plasma-treated in air for different periods of time.
